# *Cpt1a* silencing in AgRP neurons improves cognitive and physical capacity and promotes healthy aging in male mice

**DOI:** 10.1111/acel.14047

**Published:** 2023-11-22

**Authors:** Kevin Ibeas, Christian Griñán‐Ferré, Maria del Mar Romero, David Sebastián, Marianela Bastías‐Pérez, Roberto Gómez, M. Carmen Soler‐Vázquez, Sebastián Zagmutt, Mercè Pallás, Margarida Castell, Denise D. Belsham, Paula Mera, Laura Herrero, Dolors Serra

**Affiliations:** ^1^ Department of Biochemistry and Physiology, School of Pharmacy and Food Sciences Universitat de Barcelona Barcelona Spain; ^2^ Institut de Biomedicina de la Universitat de Barcelona (IBUB), Universitat de Barcelona Barcelona Spain; ^3^ Centro de Investigación Biomédica en Red de Fisiopatología de la Obesidad y la Nutrición (CIBEROBN) Instituto de Salud Carlos III Madrid Spain; ^4^ Department of Pharmacology, Toxicology and Therapeutic Chemistry, School of Pharmacy and Food Sciences Universitat de Barcelona Barcelona Spain; ^5^ Centro de Investigación en Red, Enfermedades Neurodegenerativas (CIBERNED Instituto de Salud Carlos III Madrid Spain; ^6^ Centro de Investigación Biomédica en Red de Diabetes y Enfermedades Metabólicas Asociadas (CIBERDEM) Instituto de Salud Carlos III Madrid Spain; ^7^ Institut de Recerca en Nutrició i Seguretat Alimentària (INSA‐UB), Universitat de Barcelona Santa Coloma de Gramenet Spain; ^8^ Department of Physiology, Obstetrics and Gynaecology and Medicine University of Toronto Toronto Ontario Canada; ^9^ Present address: Facultad de Salud y Ciencias Sociales Universidad de las Américas Santiago de Chile Chile

**Keywords:** aging, AgRP neurons, cognitive behavior, CPT1A, physical activity

## Abstract

Orexigenic neurons expressing agouti‐related protein (AgRP) and neuropeptide Y in the arcuate nucleus (ARC) of the hypothalamus are activated in response to dynamic variations in the metabolic state, including exercise. We previously observed that carnitine palmitoyltransferase 1a (CPT1A), a rate‐limiting enzyme of mitochondrial fatty acid oxidation, is a key factor in AgRP neurons, modulating whole‐body energy balance and fluid homeostasis. However, the effect of CPT1A in AgRP neurons in aged mice and during exercise has not been explored yet. We have evaluated the physical and cognitive capacity of adult and aged mutant male mice lacking *Cpt1a* in AgRP neurons (*Cpt1a* KO). Adult *Cpt1a* KO male mice exhibited enhanced endurance performance, motor coordination, locomotion, and exploration compared with control mice. No changes were observed in anxiety‐related behavior, cognition, and muscle strength. Adult *Cpt1a* KO mice showed a reduction in gastrocnemius and tibialis anterior muscle mass. The cross‐sectional area (CSA) of these muscles were smaller than those of control mice displaying a myofiber remodeling from type II to type I fibers. In aged mice, changes in myofiber remodeling were maintained in *Cpt1a* KO mice, avoiding loss of physical capacity during aging progression. Additionally, aged *Cpt1a* KO mice revealed better cognitive skills, reduced inflammation, and oxidative stress in the hypothalamus and hippocampus. In conclusion, CPT1A in AgRP neurons appears to modulate health and protects against aging. Future studies are required to clarify whether CPT1A is a potential antiaging candidate for treating diseases affecting memory and physical activity.

## Graphical abstract

The specific deletion of Cpt1a in AgRP neurons improves health and lifespan. AgRP neurons require CPT1A to modulate exercise performance in adult and aged mice. CPT1A in AgRP neurons regulates muscle mass and myofiber composition. Cpt1a ablation in AgRP neurons improves memory and neuroplasticity during aging.

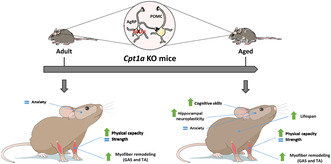

**Abbreviations**
AgRP: Agouti‐related protein
ARC: Arcuatenucleus
Atgl: Adipose triglyceride lipase
Bdnf: Brain‐derived neurotrophic factor
Cpt1a: Carnitine palmitoyl transferases 1a
CSA: Cross‐sectional area
Ddit3: DNA damage‐inducible transcript 3
DI: Discrimination Index
DRP1: Dynamin‐related protein 1
EDL: Extensor‐digitorum longus
EPM: Elevated Plus Maze
Fbxo32: F‐box protein 32, atrogin‐1
Gapdh: Glyceraldehyde‐3‐phosphatedehydrogenase
GAS: Gastrocnemius
Gdf8: Growth differentiation factor 8, myostatin
gDNA: Genomicdeoxyribonucleic acid
Hif1α: Hypoxia‐inducible factor 1 alpha
HPA: Hypothalamic‐pituitary‐adrenal axis
Hprt: Hypoxanthine phosphoribosyl‐transferase1
Hsl: Hormone‐sensitive lipase
Igf‐1: Insulin growth factor‐1
Il‐1b: Interleukin‐1b
Il‐6: Interleukin 6
KO: Knockout
MFN2: Mitofusina2
Murf1: Muscle ring finger protein 1
Musk: Muscle‐specific tyrosine kinase receptor
Myh: Myosin heavy‐chain
Ncam1: Neural cell adhesion molecule 1
NMJS: Neuromuscular junctions
NORT: Novel Object Location test
NOS2: Nitric oxide synthase 2
NPY: Neuropeptide Y
Nrf2: Nuclear factor erythroid 2–related factor 2
OFT: Open field test
OLT: Object location test
OPA1: optic atrophy 1
OXPHOS: Oxidative phosphorylation system
PBS: Phosphate‐buffered saline
Pgc1α: Peroxisome proliferator‐activated receptor γ coactivator‐1α
pCreb: cAMP‐response element‐binding protein
POMC: Pro‐opiomelanocortin
Psd95: Postsynaptic density protein 95
qRT‐PCR: Quantitative real‐time polymerase chain reaction
QUA: Quadriceps
ROS: Reactive oxygen species
Slc2a1: Glucose transporter solute carrier family 2a1
SNS: Sympathetic nervous system
Sod1: Superoxide dismutase
SOL: Soleus
TA: Tibialis anterior
Tnfα: Tumor necrosis factor alpha
Vegf: Vascular endothelial growth

## 1 INTRODUCTION

Aging is a natural and irreversible physiological process that involves a wide variety of molecular and cellular damage, leading to a gradual reduction in physical and cognitive abilities and an increased risk of health problems (Sui et al., 2021). Loss of brain function is characterized by a reduced capacity for signal transduction between cells that accelerates the development of neurodegenerative diseases, such as Alzheimer's and Parkinson's diseases (Esopenko & Levine, 2015). Skeletal muscle is highly affected by aging and displays increased muscle atrophy and reduced muscle strength and mitochondrial activity (Beaudart et al., 2015; Gouspillou et al., 2014). Physical activity has emerged as a potential therapy to improve the mental and muscle decline showed during aging (Bai et al., 2022; Ingold et al., 2020; Jakicic et al., 2018; Zhidong et al., 2021). Exercise delays the development of dementia and contributes positively to memory in middle‐aged and older people, increasing quality of life (Chekroud et al., 2018; Colcombe et al., 2003; Hart & Buck, 2019; Markov et al., 2022).

Physical activity represents an enormous challenge to whole‐body homeostasis. It causes metabolic disruption in response to an increased energy demand by skeletal muscle (Pedersen, 2019). The central nervous system, specifically the hypothalamus, plays a crucial role in the coordination of this response delivering efferent outputs via endocrine and nervous systems to maintain energy balance (Ibeas et al., 2021). The hypothalamus is organized into distinct neuronal nuclei with specific physiological functions. The arcuate nucleus (ARC) is a key region controlling feeding behavior and the maintenance of energy homeostasis. It is composed by two well‐characterized neuronal populations with antagonistic effects on appetite: (1) neurons that express anorexigenic peptides, such as pro‐opiomelanocortin and cocaine and amphetamine regulated transcript, with appetite‐suppressing functions (Albarado et al., 2004; Padilla et al., 2010; Yaswen et al., 1999); and (2) neurons that coexpress orexigenic peptides, including AgRP and neuropeptide Y (NPY), with an appetite‐stimulating role (Aponte et al., 2011; Krashes et al., 2011; Luquet et al., 2005), and reviewed in Deem et al., 2022.

AgRP neurons have been extensively studied over the last decades, and new data has emerged to suggest that AgRP neurons play a role in the regulation of metabolism during physical activity. Recent studies have demonstrated AgRP neuronal activity is altered during exercise (Landry et al., 2022; MacKay et al., 2019; Miletta et al., 2020). Acute‐ to moderate‐intensity treadmill exercise increases AgRP activity and subsequent food intake in fed mice (Bunner et al., 2020). In addition, AgRP neurons can modulate physical performance via the sympathetic nervous system (SNS) and catecholamine secretion. The activation of adrenergic pathways stimulates lipolysis, gluconeogenesis, and glycogenolysis in peripheral tissues to provide energy for muscle activity (Droste et al., 2003; Kruk et al., 2020; Miletta et al., 2020). Collectively, these data indicate a close crosstalk between AgRP neurons and skeletal muscle in the maintenance of physical activity.

Hypothalamic fatty acid metabolism plays an important role in the control of energy balance (Loftus et al., 2000; Mera et al., 2014; Obici et al., 2002; Seoane‐Collazo et al., 2018; Swierczynski et al., 2008). Specifically, CPT1A catalyzes the rate‐limiting step in the transport of long‐chain fatty acids as acyl‐CoA from the cytoplasm to the mitochondrial matrix to be oxidized. This enzyme has emerged as an important regulator of AgRP neuronal function in the maintenance of energy balance. AgRP neuron‐specific deletion of *Cpt1a* reduced food intake, body weight, and increased energy expenditure in a sex‐dependent manner (Zagmutt et al., 2023). However, the role of CPT1A in AgRP neurons in aging and exercise remains unexplored. In the present study, we analyzed the effect of *Cpt1a* deletion in AgRP neurons on exercise and aging. Our results have demonstrated that adult and aged *Cpt1a* KO mice exhibited improved exercise performance via myofiber remodeling in the gastrocnemius (GAS) and tibialis anterior (TA) muscles. At central level, *Cpt1a* KO mice showed reduced expression of oxidative stress and inflammatory markers in the hippocampus and hypothalamus, which results in an improvement in memory and cognition. In addition, aged *Cpt1a* KO mice displayed an increased lifespan. These results highlight CPT1A as a target enzyme for the treatment of several disorders associated with muscle damage or memory loss.

## 2 METHODS AND EXPERIMENTAL PROCEDURES

### 2.1 Animal models

C57BL/6J background male and female *Cpt1a* KO (*Cpt1a* ^flox/flox^ and *Agrp* ^CreERT2^) and control littermate (*Cpt1a* ^+/+^ and *Agrp* ^CreERT2^) inducible mice were created previously in our laboratory (Mir et al., 2018; Zagmutt et al., 2023). The genotyping is performed using specific primers against *Cpt1a* Flox and AgRP CreERT2 regions described in Table S1.Both *Cpt1a* KO and control mice were injected with tamoxifen dissolved in corn oil to induce Cre recombinase expression, as described in (Zagmutt et al., 2023). All the studies used age‐matched littermates. Physical, behavioral, and cognitive tests were performed on 4‐month‐old (adult) and 18‐month‐old (aged) mice. All tests were conducted during the light cycle. Mice were kept under standard conditions with ad libitum access to a standard chow diet (SAFE, SAFE® 105) and water in a HEPA filtered room at a controlled temperature (22 ± 2°C) and humidity (50%–60%). All animals were maintained on a 12 h light/dark cycle and daily health monitoring. All the experiments were approved by the Animal Experimentation Ethics Committee of the University of Barcelona (CEEA‐UB), procedure number CEEA 10994, obtained from the Government of Catalonia.

### 2.2 Treadmill fatigue test

The treadmill fatigue test determines the endurance capacity of rodents. The protocol followed was described by Dougherty et al., 2016. Prior to the test, animals were trained using a treadmill Exer 3/6 (Columbus) for 3 consecutive days. The inclination was 10% and the intensity and frequency of the electric grid were 1.22 mA and 2 Hz, respectively. The exhaustion protocol was: 5 min rest, 30 min at 10 m/min, 10 min at 11 m/min, 10 min at 12 m/min, 10 min at 13 m/min, 5 min at 14 m/min and then, the speed was increased 1 m/min every 5 min until the criterion for exhaustion was met, which is defined as spending 5 consecutive seconds on the electric grid and failing to continue running.

### 2.3 Open field test (OFT)

The OFT evaluates locomotor activity, anxiety, and willingness to explore. Before the test, mice were acclimatized to the smell and handling of the researcher to avoid changes in behavior. Mice were placed at the center of the 50 × 50 × 25 cm white polywood box and allowed to explore for 10 min. Mouse behavior and locomotor activity were recorded and analyzed using SMART software (version 3.0).

### 2.4 Elevated plus maze test (EPMT)

The EPMT measures mouse anxiety‐related behavior. The maze is composed by two open arms (5 × 30 × 1 cm) and two close arms (5 × 30 × 15 cm). Prior to the test, mice were acclimatized to the smell and handling of the researcher to avoid behavior alterations. Mice were placed in the center area of the maze and allowed to explore for 5 min. Mouse behavior and locomotor activity were recorded and analyzed using SMART software (version 3.0).

### 2.5 Rotarod test

Mice motor coordination and balance were examined using the rotarod according to the protocol described by Deacon, 2013a, 2013b. Animals were placed on the rotating rod (Columbus Instruments), facing away from the direction of rotation. The speed started at 4 rpm and accelerated at 1 rpm/8 s to a maximum of 40 rpm. Three continuous trials were performed with 5 min of rest, and the best time obtained was used for data analysis. Data are presented as latency to fall (s).

### 2.6 Kondziela's inverted screen test

The Kondziela's inverted screen test evaluates the muscle strength in the four limbs (Deacon, 2013b). The inverted screen consists of a 15 × 10 cm rectangle of wire mesh surrounded by a 3 cm deep wood base to prevent mice from attempting to climb on the upper side. The structure is designed to fit correctly within the experimental cage, which is 25% filled with preheated water. Mice were placed in the center of the inverted screen and the time achieved until falling into the water was measured. Two continuous trials were performed with a 20 min rest period between trials. Data were analyzed and expressed as holding impulse (N·s) considering the body weight and the gravity force.

### 2.7 Novel object recognition test (NORT)

The NORT evaluates the short‐ and long‐term recognition memory. The apparatus consisted of a two black polyvinyl chloride arms (25 × 20 × 5 cm) at 90° to each other. Objects used in the test were 10 cm high and not frightening for mice. The familiarization session was conducted for 3 days, in which mice were individually habituated to the apparatus for 10 min. On day 4, two identical objects (A) were placed at the end of each arm and animals were allowed to explore freely for 10 min (Trial 1). Two hours later, a second trial (Trial 2) was performed replacing one of the previous objects (A) with a novel object of distinct colour and shape (B). Twenty‐four hours later, mice were assessed again (Trial 3) using one of the previous objects (A) and a novel object of a distinct color and shape (C). To avoid object preferences bias, the position of the novel object and objects used were randomized among mice. The criterion of exploration was defined as pointing the nose toward the object at a distance ≤2 cm and/or touching the object with the nose. Turning or sitting around the object was not considered exploration. Mouse exploratory behavior was recorded, and recognition memory was evaluated using the discrimination index (DI), defined as: (time spent in the novel object‐time spent in old object)/(total time exploring both objects). A value of zero indicates that mice explore both objects without any preference.

### 2.8 Object location test (OLT)

The OLT determines the spatial memory. This test was performed using the same apparatus as the OFT, but one of the walls was black. The training session consisted of 3 days of adaptation. On day 1, mice were habituated to the empty open field arena for 10 min. On day 2, two objects (A) were placed in front of the black wall, equidistant from each other and the wall. The objects used were identical, 10 cm high and not frightening for mice. Animals were placed into the apparatus and allowed to explore for 10 min. On day 3, one of the objects was moved to a novel position (A') to assess their spatial memory. Explorative behavior was recorded using a camera and cognitive ability was analyzed using the DI. A value of zero indicates that mice investigate both objects without any preference.

### 2.9 Euthanasia and tissue collection from mice

To obtain tissue and blood samples, mice were fasted for 2 h, anesthetized with 4% isoflurane (Piramal Healthcare), and maintained by continuous inhalation of 2% isoflurane with the machine (Combi‐Vet Rothacher Medical). Tissue collection is described in the Appendix S1. Samples were obtained and stored immediately at −80°C.

### 2.10 Histological analysis

Tissue samples were paraffin‐embedded, cut into 4 μm sections and stained with H&E. Images were taken using a Leica DM IL LED microscope (Leica). The processing of images and measurement of fiber size were performed using Image J software (Version 1.8.0, Schneider et al., 2012).

### 2.11 Muscle cross‐sections preparations and immunostaining

Cryopreserved muscles were cut into 15 μm sections using a Leica CM3050S (Leica) cryostat and collected onto SuperFrost positively charged slides (Thermo Scientific). TA and GAS sequential microsections, comprising clear red and white muscle regions, were collected 1 mm from the tendon. Immunostaining techniques are described in the Appendix S1. A list of primary and secondary antibodies used is provided in Table S2.

### 2.12 Total RNA extraction and quantitative real‐time PCR (qRT‐PCR)

RNA was isolated and qRT‐PCR is performed as described in the Appendix S1. The primers used in this study are listed in Table S1.

### 2.13 Statistical analyses

Statistical analyses were determined using GraphPad Prism 9 software (GraphPad software, Version 9.3.2). A two‐tailed Student's *t*‐test was used to compare two groups (KO and control) with a specific variable. Two‐way ANOVA followed by Sidak's multiple comparisons test was used when two variables were compared between two or more groups. In the lifespan study, data were analyzed using the Gehan‐Breslow‐Wilcoxon survival curves test. Outliers were excluded following the ROUT test. Data were expressed as mean ± standard error of mean (SEM). Significant differences were considered when the level of confidence was >95% (*p* < 0.05). The number of animals/samples is specified in each figure legend.

## 3 RESULTS

### 3.1 *Cpt1a* deletion in AgRP neurons protects against aging

**FIGURE 1:**
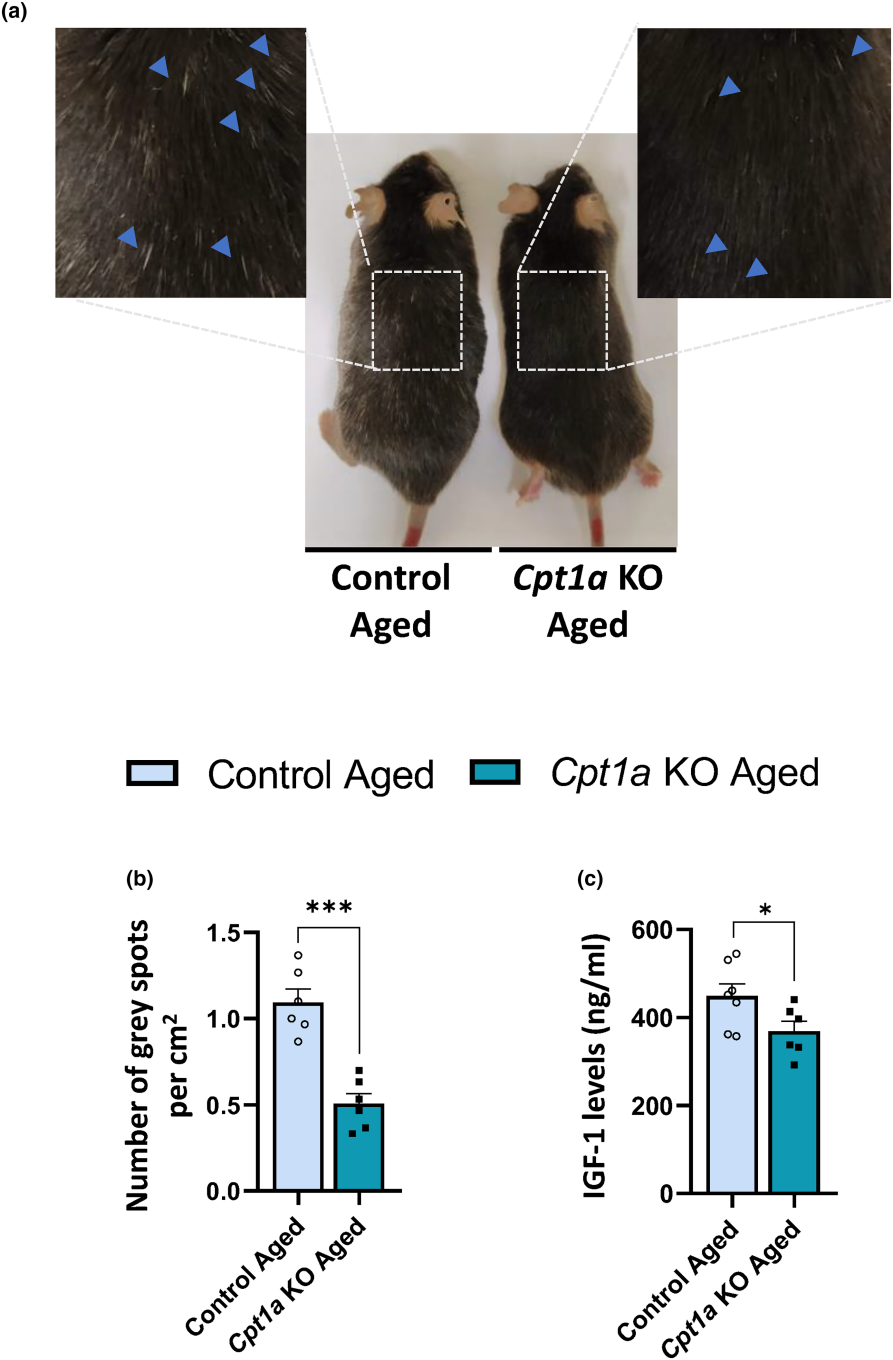
Phenotype of aged *Cpt1a* KO male mice. (a) Representative images of 20‐month‐old control and *Cpt1a* KO mice. (b) Quantification of gray hair pixels per cm^2^ on mice dorsal skin. (c) Plasma IGF‐1 levels in aged mice. (d) Survival of mice. *p < 0.05; ***p < 0.001; using two‐tailed Student's *t*‐test. (*n* = 6–9).

*Cpt1a* KO and control male mice were analyzed during aging progression. We observed that 20‐month‐old *Cpt1a* KO mice showed a 50% reduction in the number of gray spots per area (Figure 1a,b), suggesting a protection against aging. We then analyzed whether lifespan was increased in *Cpt1a* KO mice. It has been previously demonstrated that a reduction in insulin growth factor 1 (IGF‐1) levels is associated with a decrease in growth hormone signaling and increased lifespan (Junnila et al., 2013; K. Mao et al., 2018). Plasma IGF‐1 levels in aged *Cpt1a* KO male mice were significantly decreased compared with control mice (Figure 1c). A small cohort of mice was used to explore whether *Cpt1a* KO male mice could impact lifespan. The median lifespan was extended in *Cpt1a* KO mice (29.6 months) compared to control mice (25.1 months) (Figure S1a). To support these results and rule out possible toxic effects of Cre expression in AgRP neuronal viability, we analyzed expression of the neuropeptide AgRP in the ARC of overnight‐fasted control, *Cpt1a* KO, and noninduced control‐TMX aged mice (Figure S1b,c). We observed no differences in AgRP expression between the three groups of mice. These results clearly indicate that the expression of Cre recombinase does not affect the viability of the neurons and the expression of AgRP neuropeptide, although we cannot rule out that it could affect other functions of the AgRP neurons. Altogether, these results could suggest a key role of CPT1A enzyme in AgRP neurons as a modulator of aging.

### 3.2 Adult and aged *Cpt1a* KO male mice show improved physical performance without changes in strength and anxiety‐like behavior

**FIGURE 2:**
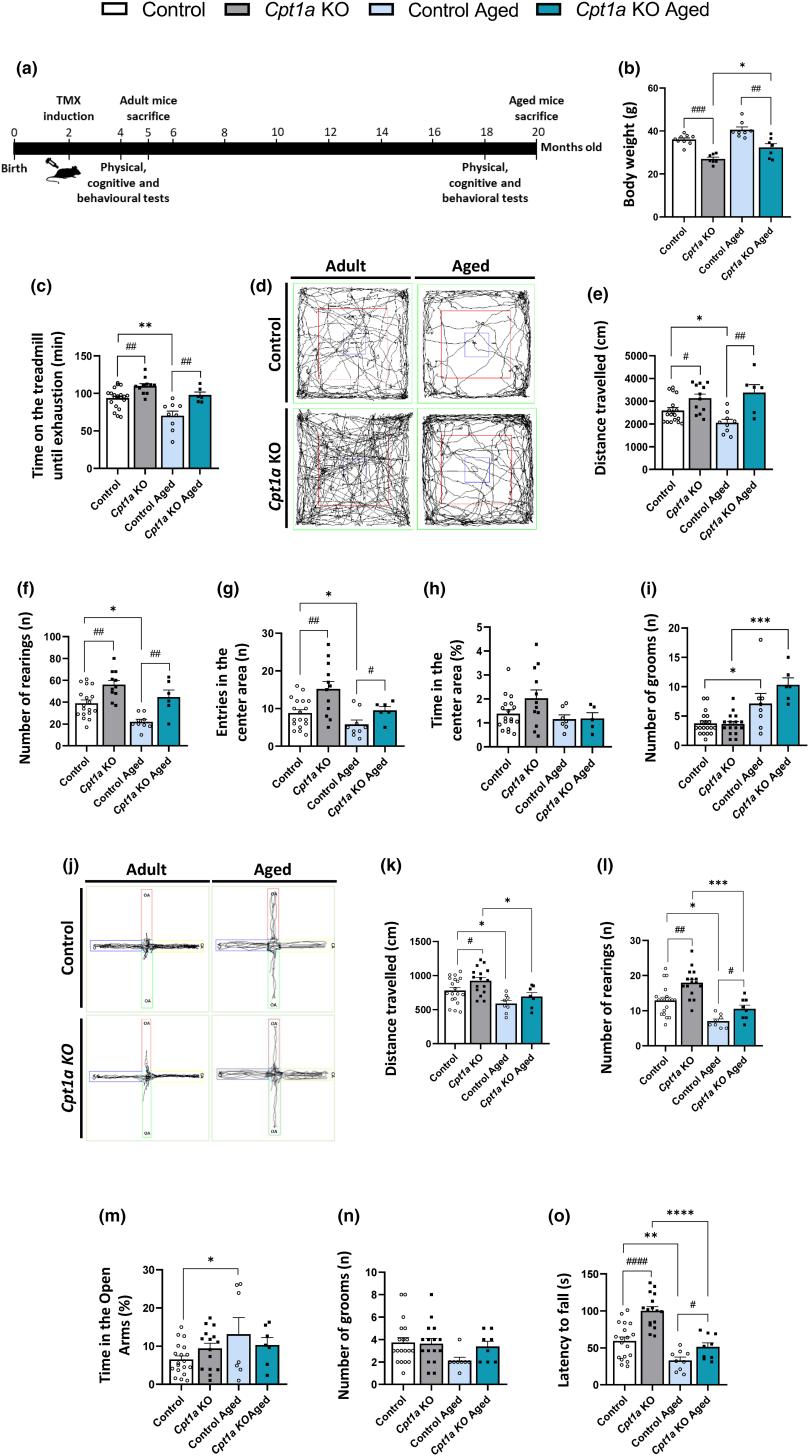
CPT1A in AgRP neurons is involved in exercise performance in adult and aged male mice. (a) Experimental time course. (b) Body weights of 5 and 20‐month‐old mice. (c) Treadmill exhaustion test. (d–i) OFT results. Representative diagrams of the distance travelled by mice (d) and their quantification (e). Number of rearings (f) and entries in the center area of the open field arena (g). Behavior: time spent in the center of the structure (h) and grooming (i). (j–m) EPMT results. Representative diagrams of EPMT (j), locomotor activity quantification (k), and exploratory capacity (l). Anxiety‐like behavior: time spent in the open arms (m), and emotional behavior: number of grooms (n). (o) Rotarod test analysis. ^# or^ *p < 0.05; ^## or^ **p < 0.01; ***p < 0.001; using two‐way ANOVA Sidak's multiple comparisons test. (*n* = 14–21 adult and *n* = 6–9 aged mice). *Indicates adult versus aged. ^#^Indicates control versus *Cpt1a* KO.

We examined whether *Cpt1a* KO mice were protected from the aging decline in physical and cognitive abilities. We first examined the effect of *Cpt1a* deletion in AgRP neurons on physical capacity in adult (5‐month‐old) and aged (20‐month‐old) male mice (Figure 2). The time course followed is described in Figure 2a. Both adult and aged *Cpt1a* KO mice showed reduced body weight compared with the control mice (Figure 2b). Adult and aged male *Cpt1a* KO mice were more active than the control mice in the locomotion tests. In the endurance test, the time running on the treadmill until exhaustion was increased in adult *Cpt1a* KO male mice compared with control mice; however, importantly, the decrease observed in the aged control mice was not observed in aged *Cpt1a* KO mice that instead maintained the same endurance as adult male mice (Figure 2c). In the OFT, adult male *Cpt1a* KO mice travelled enhanced distance compared with control mice (Figure 2d,e). This was also observed in aged *Cpt1a* KO male mice, where they exhibit a 60% improvement in locomotor activity compared with aged control mice, indicating that aging does not affect the locomotor capacity of *Cpt1a* KO mice. Adult and aged male *Cpt1a* KO mice showed an increase in exploratory capacity compared with control mice, as measured by the number of rearings (Figure 2f) and entries into the OFT center area (Figure 2g). No differences were observed in anxiety behavior, as measured by the proportion of time spent in the center area of the open field arena (Figure 2h). Furthermore, no changes in emotional conduct, as measured by the number of grooms, were observed (Figure 2i).

The EPM test confirmed the increased locomotion activity of adult *Cpt1a* KO male mice respect to the control mice (Figure 2j,k). This improvement was not observed in aged *Cpt1a* KO male mice. Both adult and aged *Cpt1a* KO mice showed enhanced exploratory capacity (Figure 2l) without alterations in anxiety and emotional behavior, as analyzed by the percentage of time that mouse was in the open arms of the EPM structure and the number of grooms (Figure 2m,n). Motor coordination was analyzed using the rotarod test. Aged control and *Cpt1a* KO male mice had a reduction in the coordination due to aging, as compared to adult mice; however, adult and aged *Cpt1a* KO mice spent more time on the rod than control mice (Figure 2o). *Cpt1a* KO in adult and aged male mice showed no impact on strength capacity in the Kondziela's inverted screen (Figure S2a) and weight tests (Figure S2b). Since *Cpt1a* KO male mice showed reduced body weight, the muscle strength test was corrected to body weight.

Adult female *Cpt1a* KO mice were also studied in response to exercise. No differences were observed in endurance (Figure S2c) and strength (Figure S2d) between female *Cpt1a* KO mice and control highlighting sex‐dependent differences in the physical activity. Since females showed no changes in physical activity, our subsequent experiments were only performed in males. Taken together, these results demonstrate that the specific deletion of *Cpt1a* in AgRP neurons enhances physical activity and suggest an important role of CPT1A enzyme in AgRP neurons in the delay of the decline in physical performance associated with age in males.

### 3.3 CPT1A in AgRP neurons regulates GAS muscle mass and myofiber composition

**FIGURE 3:**
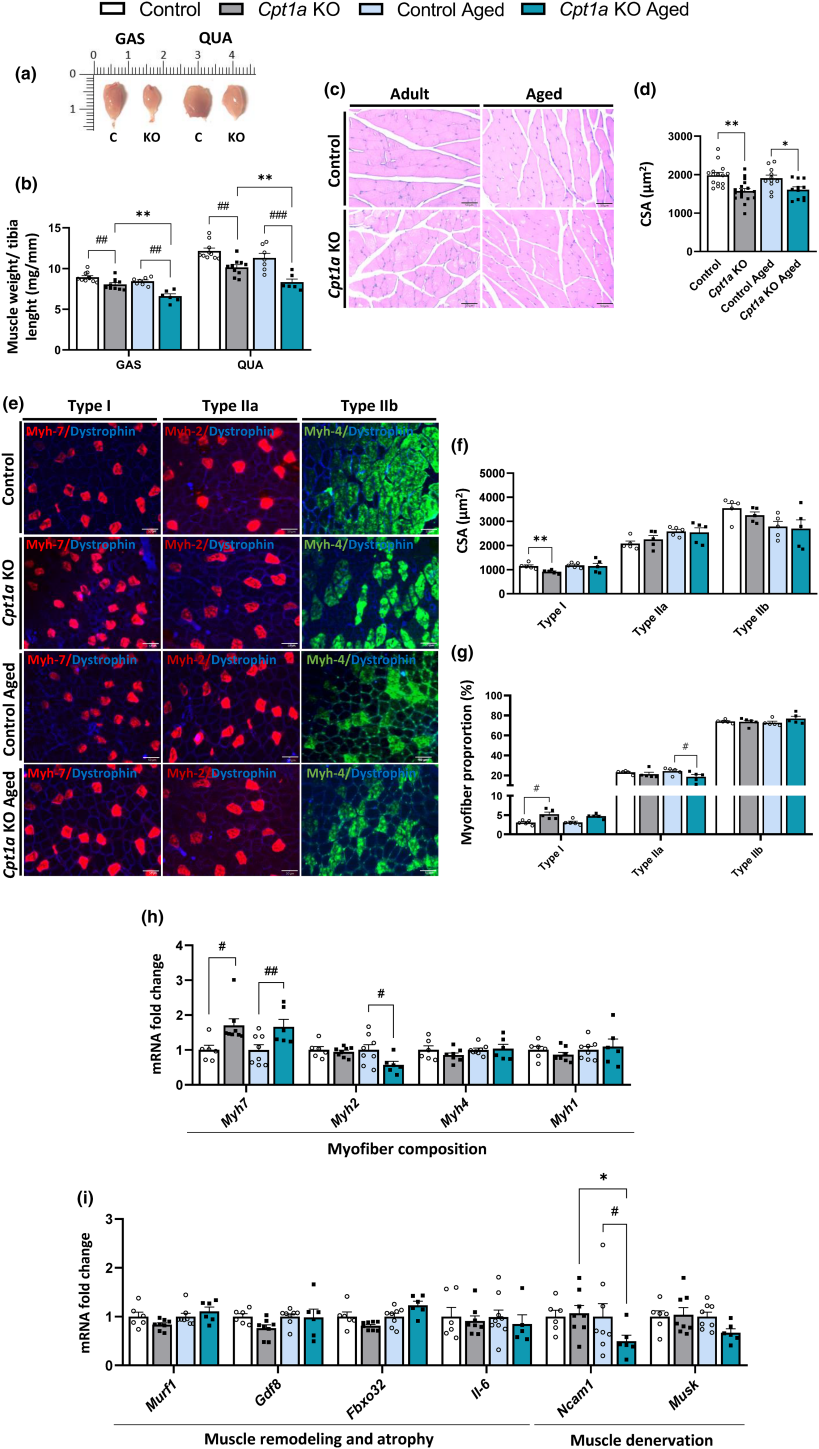
Muscle mass and myofiber composition of the GAS muscle. (a) Representative images of the GAS and QUA muscles of adult *Cpt1a* KO and control mice. (b) GAS and QUA muscle mass. (c,d) Adult and aged mice CSA of GAS muscle. GAS cross‐sections stained with H&E (c) and CSA quantification (d). (e) Analysis of GAS myofiber composition by immunofluorescence. (f,g) CSA quantification (f) and percentage of fiber types (g). (h,i) mRNA levels of genes that encode for the myofiber types (h), muscle atrophy, and denervation in the GAS muscle (i). ^# or^ *p < 0.05; ^## or^ **p < 0.01; ^###^ *p* < 0.001; ****p < 0.0001 using two‐way ANOVA Sidak's multiple comparisons test (b, d–g) and (i–j) and two‐tailed Student's *t*‐test (h). (*n* = 5–17 adult and *n* = 5–11 aged mice). *Indicates adult versus aged. ^#^Indicates control versus *Cpt1a* KO.

Muscle mass and fiber morphology in the skeletal muscle was studied to explore the differences observed in physical performance. Adult and aged *Cpt1a* KO male mice exhibited a reduction in GAS and quadriceps (QUA) muscle mass compared with the control group (Figure 3a,b), indicating a decrease in muscle mass. This reduction is generally associated with a reduction in the CSA. Adult *Cpt1a* KO mice showed a 16% reduction in the GAS CSA (Figure 3c,d), which resulted in increased number of 500–1000‐μm^2^ myofibers and reduced number of 2500–3000 and 3500–4000‐μm^2^ fibers (Figure S3a). Like adult mice, aged *Cpt1a* KO mice exhibited a reduction in CSA (Figure 3d), characterized by a reduction in the quantity of 2500–3000 and 3500–4000‐μm^2^ muscle fibers (Figure S3a).

To understand how *Cpt1a* silencing in AgRP neurons alters muscle fiber size, the GAS myofiber composition was determined in adult and aged mice. Immunohistochemical analysis of GAS cross‐sections demonstrated a decreased area of type I fibers in adult *Cpt1a* KO mice (Figure 3e,f) and an increase in the percentage of type I myofibers in adult *Cpt1a* KO mice compared with control mice (Figure 3g). Additionally, no changes were observed for CSA and distribution of type II fibers in the GAS muscle of adult and aged *Cpt1a* KO male mice. To confirm the increase in the number of type I myofibers in the GAS the mRNA levels of different myosin heavy chain (*Myh*) genes related to myofiber types were evaluated (Figure 3h). Consistent with the histological results, the mRNA levels of *Myh7*, which encodes oxidative fibers, were boosted in adult and aged *Cpt1a* KO mice compared with the control mice which supports a rise of oxidative fibers I (Figure 3h). Aged *Cpt1a* KO mice exhibited significantly decreased mRNA levels of *Myh2*, which encodes the most oxidative type II fiber, type IIa. These data suggest a muscle fiber remodeling from type IIa to type I myofibers in *Cpt1a* KO mice.

Despite changes in the myofiber composition of *Cpt1a* KO mice, no alterations were observed in mRNA levels of genes involved in muscle atrophy, such as muscle ring finger protein 1 (*Murf1*), myostatin (*Gdf8*), and F‐box protein 32 (*Fbxo32*) as well as levels of interleukin 6 (*Il‐6*), which is a myokine with pleotropic functions that is associated with induction stimulation of muscle hypertrophy and myogenesis (Figure 3i). In addition, adult AgRP *Cpt1a* KO male mice did not exhibit significant differences in neural cell adhesion molecule 1 (*Ncam1*) and muscle‐associated receptor tyrosine kinase (*Musk*), two genes related to muscle denervation and neuromuscular junctions (NMJs). However, aged *Cpt1a* KO male mice had a significant decrease in mRNA levels of *Ncam1*, which could suggest a lower muscle denervation during the progression of age (Figure 3i).

### 3.4 Deletion of *Cpt1a* in AgRP neurons promotes myofiber remodeling toward oxidative fibers in the TA muscle in adult and aged male mice

**FIGURE 4:**
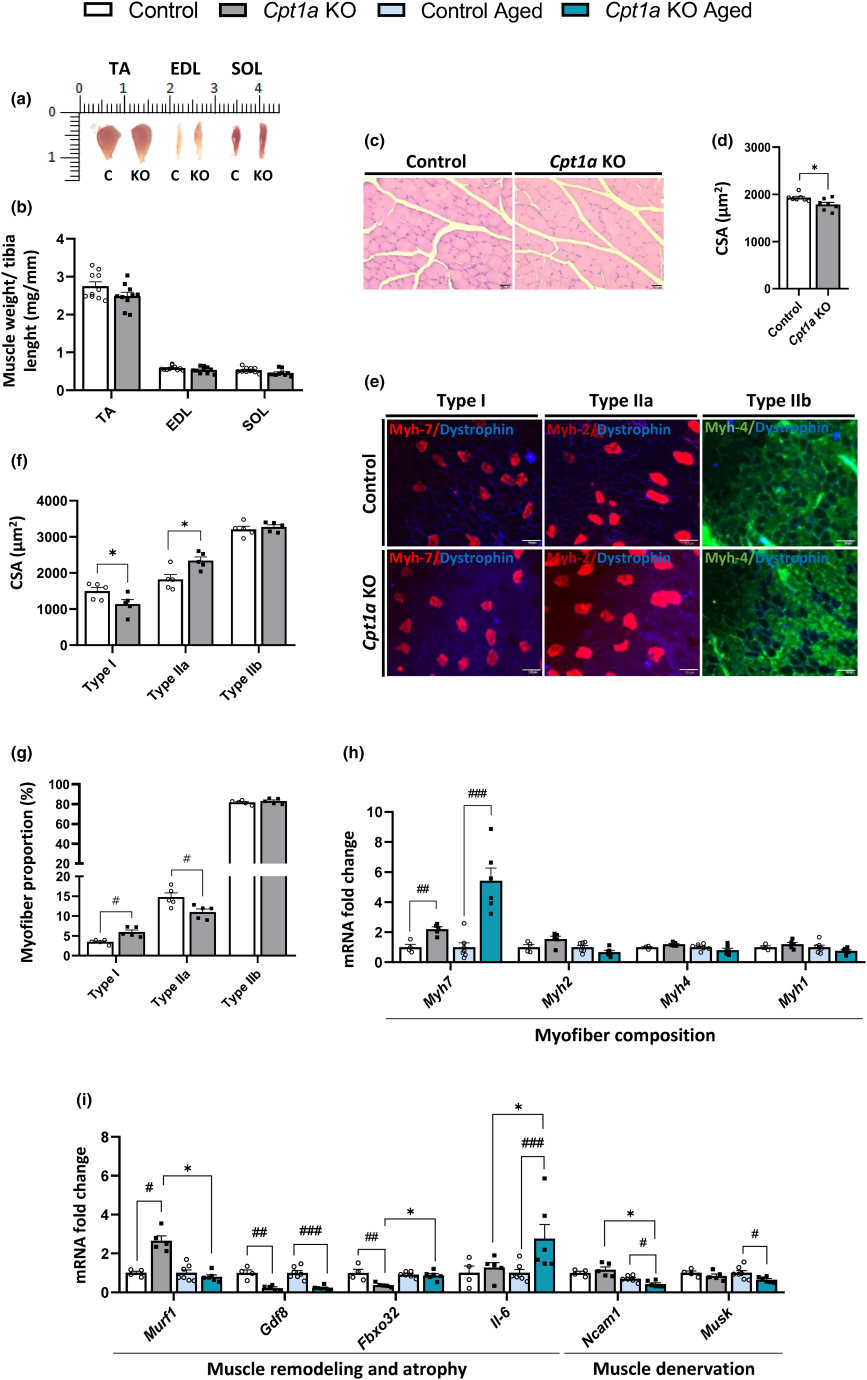
Analysis of TA muscle myofiber composition in adult and aged male mice. (a) Representative images of TA, EDL, and SOL muscles in adult mice. (b) Muscle mass of TA, EDL, and SOL muscles. (c,d) Myofiber CSA of adult TA muscle. TA cross‐sections stained with H&E and (c) and CSA quantification (d). (e–g) Analysis of TA myofiber composition of adult mice. Quantification of the CSA of TA muscle (f) and percentage of fiber types in adult TA muscles (g). (h,i) mRNA levels of genes that encode myofiber types (h), muscle atrophy, and denervation in the TA muscle (i). ^# or^ *p < 0.05; ^##^ *p* < 0.01; ^###^ *p* < 0.001 using two‐tailed Student's *t*‐test (a–g) and two‐way ANOVA Sidak's multiple comparisons (h,i). (*n* = 5–10 adult and *n* = 5 aged mice). *Indicates adult versus aged. ^#^Indicates control versus *Cpt1a* KO.

The muscle mass of adult *Cpt1a* KO male mice was also examined in the smallest muscles, including the TA, extensor digitorum longus (EDL), and soleus (SOL). However, no differences in the size of these muscles were detected (Figure 4a,b). Even though we did not observe differences in the TA muscle mass, adult *Cpt1a* KO mice showed a 10% reduction in the CSA of the TA muscle compared with the control mice (Figure 4c,d). The myofiber distribution revealed the same pattern described in the GAS muscle, with *Cpt1a* KO mice demonstrating an increased number of 500–1000‐μm^2^ fibers (Figure S2b). Accordingly, adult mice lacking *Cpt1a* in AgRP neurons exhibited a decreased CSA of type I myofibers and an increased CSA of type IIa fibers (Figure 4e,f), with no changes in type IIb myofibers between groups. In addition, an increase in the number of type I fibers was observed in adult *Cpt1a* KO mice, whereas the number of type IIa fibers decreased compared with the control mice (Figure 4g). Both adult and aged *Cpt1a* KO mice showed an increase in *Myh7* mRNA levels in this muscle (Figure 4h). No differences were observed in *Myh2*, *Myh4*, and *Myh1* mRNA levels in adult and aged *Cpt1a* KO mice compared with the control mice.

In adult *Cpt1a* KO mice, mRNA levels of the atrophy gene, *Murf‐1*, were increased, whereas there was a significant reduction in mRNA levels of *Gdf8* and *Fbxo32*, two key factors involved in the muscle atrophy process (Figure 4i). Similarly, aged *Cpt1a* KO mice had dramatically reduced mRNA levels of *Gdf8*, suggesting inhibition of TA atrophy and enhanced muscle strength. In addition, myokine *Il‐6* was augmented in aged *Cpt1a* KO mice, indicating a better adaptation to physical activity. Aged mice lacking *Cpt1a* in AgRP neurons could have an improvement in the TA NMJs via a reduction in *Ncam1* and *Musk* mRNA levels (Figure 4i).

We also examined the myofiber composition of the EDL and SOL muscles in adult mice. However, no alterations in the mRNA levels of the specific *Myh* genes were observed in the glycolytic EDL muscle (Figure S3c) and the oxidative muscle SOL (Figure S3d). Taken together, these findings suggest that the specific deletion of *Cpt1a* in AgRP neurons may improve exercise performance although it is unclear whether myofiber remodeling is the cause or consequence of this improvement.

### 3.5 *Cpt1a* KO male mice exhibit increased mitochondrial content, altered angiogenesis maintaining the oxidative capacity in the TA and GAS muscles

**FIGURE 5:**
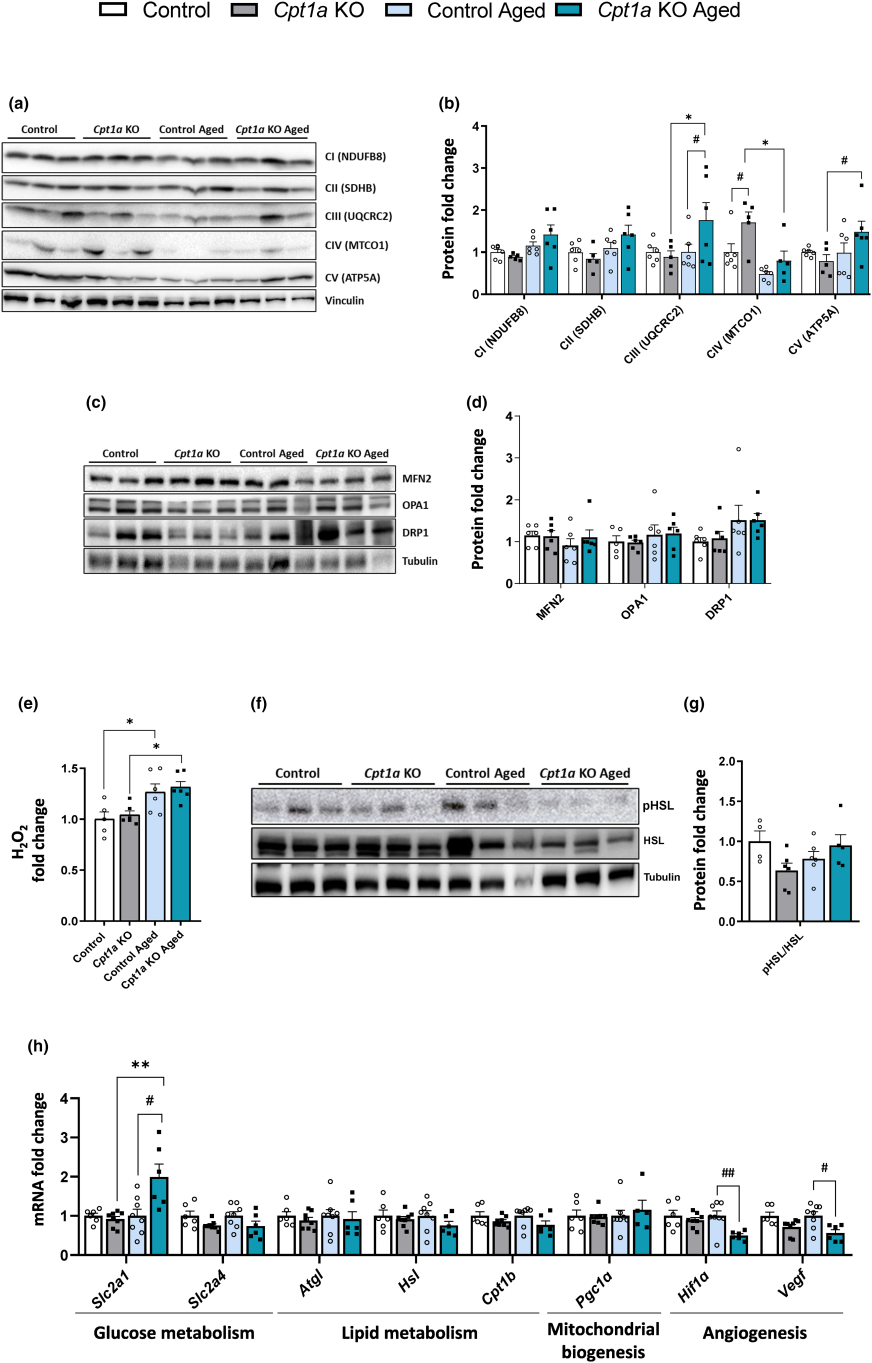
Analysis of mitochondrial content, mitochondrial dynamics, metabolism, and angiogenesis in GAS muscle. (a,b) Analysis of the mitochondrial content. Representative Western blot of the mitochondrial OXPHOS proteins (a). Quantification (b). (c,d) Analysis of mitochondrial dynamics. Representative Western blot of mitochondrial dynamics protein markers (c). Quantification (d). (e) Analysis of ROS (H_2_O_2_ levels). (f,g) Analysis of the protein marker of lypolisis. Representative Western blot of pHSL(Ser565) and total HSL proteins (f). Quantification (g). (h) mRNA levels of genes related to glucose and lipid metabolism, mitochondrial biogenesis and angiogenesis. ^# or^ *p < 0.05; ^## or^ **p < 0.01; ^###^ *p* < 0.001; using two‐tailed Student's *t*‐test (f,g) and two‐way ANOVA Sidak's multiple comparisons (a–d) and g. (*n* = 4–9 adult and = 6–9 aged mice) *Indicates adult versus aged. ^#^Indicates control versus *Cpt1a* KO.

To determine whether the variations in myofiber composition of the GAS and TA muscles could modify the mitochondrial content, we analyzed levels of the mitochondrial oxidative phosphorylation (OXPHOS) I–V proteins. Protein levels of complex III and V increased in the GAS of aged *Cpt1a* KO mice, while complex IV protein levels were only increased in adult *Cpt1a* KO mice (Figure 5a,b). These results suggest an increase in mitochondrial content in the GAS muscle in aged *Cpt1a* KO mice. Mitochondrial dynamics may be involved in the changes in mitochondrial content, but no changes were observed in the protein levels of mitochondrial dynamics mitofusin 2 (MFN2), optic atrophy 1 (OPA1), and dynamin‐related protein (DRP1) in both adult and aged mice (Figure 5c,d). No changes were observed in mRNA levels of peroxisome proliferator‐activated receptor γ coactivator 1α (*Pgc1α*), which is a key regulator of mitochondrial biogenesis and oxidative capacity (Figure 5h). We also analyzed reactive oxygen species (ROS) production by measuring H_2_O_2_ levels. Aged mice showed increased production of ROS compared with adult mice; however, no differences were observed between control and *Cpt1a* KO mice (Figure 5e). No differences were observed in the mRNA profiles of lipid and glucose metabolism among adult mice (Figure 5h). However, aged *Cpt1a* KO mice exhibited a significant increase in the mRNA levels of glucose transporter 1 (*Slc2a1)*, but there were no changes in genes associated with lipolytic pathways, including adipose triglyceride lipase (*Atgl*), hormone‐sensitive lipase (*Hsl*), and *Cpt1b*. No changes were observed in the phosphorylated levels of the hormone‐sensitive lipase (pHSL) protein (Figure 5f–h). Additionally, only aged *Cpt1a* KO mice had a decrease in mRNA levels of genes involved in angiogenesis, suggesting that under hypoxic conditions, aged *Cpt1a* KO mice do not require an additional source of oxygen because they have enough to perform physical activity (Figure 5h).

In the TA muscle, protein levels of OXPHOS complex V increased, whereas protein complex IV showed a significant reduction in aged *Cpt1a* KO mice (Figure S4a,b). Regarding mitochondrial dynamics, no changes were observed in the protein levels of MFN2, OPA1, and DRP1 in *Cpt1a* KO mice (Figure S4c,d). In addition, no changes in mRNA levels of *Pgc1α* were observed (Figure S4f). ROS content is increased in aged mice but no differences were observed between control and *Cpt1a* KO mice (Figure S4e). Deletion of *Cpt1a* in AgRP neurons significantly increased mRNA levels of *Slc2a1*. Although we observed an increase of mRNA levels of genes involved in lipolytic pathways, including *Atgl* and *Hsl*, the pHSL protein was not detected in *Cpt1a* KO adult mice suggesting that the lipolytic pathway is not activated (Figure S4f–h). These alterations were not maintained in aged mice, suggesting a different adaptation during the progression of aging. In addition, both adult and aged *Cpt1a* KO mice exhibited a reduction in mRNA levels of hypoxic inducible factor 1α (*Hif1α*) and vascular endothelial growth factor (*Vegf*) (Figure S4h). These results demonstrate that, despite similarities in myofiber remodeling, each muscle can individually adapt its metabolism and mitochondrial content.

To determine whether the changes observed in the muscle and physical performance of *Cpt1a* KO mice were consequences of alterations to the hypothalamic–pituitary–adrenal axis (HPA), we measured plasma levels of catecholamines and cortisone. Adult and aged male mice lacking *Cpt1a* in AgRP neurons showed no changes in plasma levels of these hormones (Figure S4i,j). Besides, no changes were observed in plasma glucose levels (Figure S4k) after 2 h fasting. These findings indicate that catecholamines and cortisone do not influence the physical performance of *Cpt1a* KO mice.

### 3.6 Aged Cpt*1a* KO male mice maintain cognition, neuroplasticity, and reduced oxidative stress during aging progression

**FIGURE 6:**
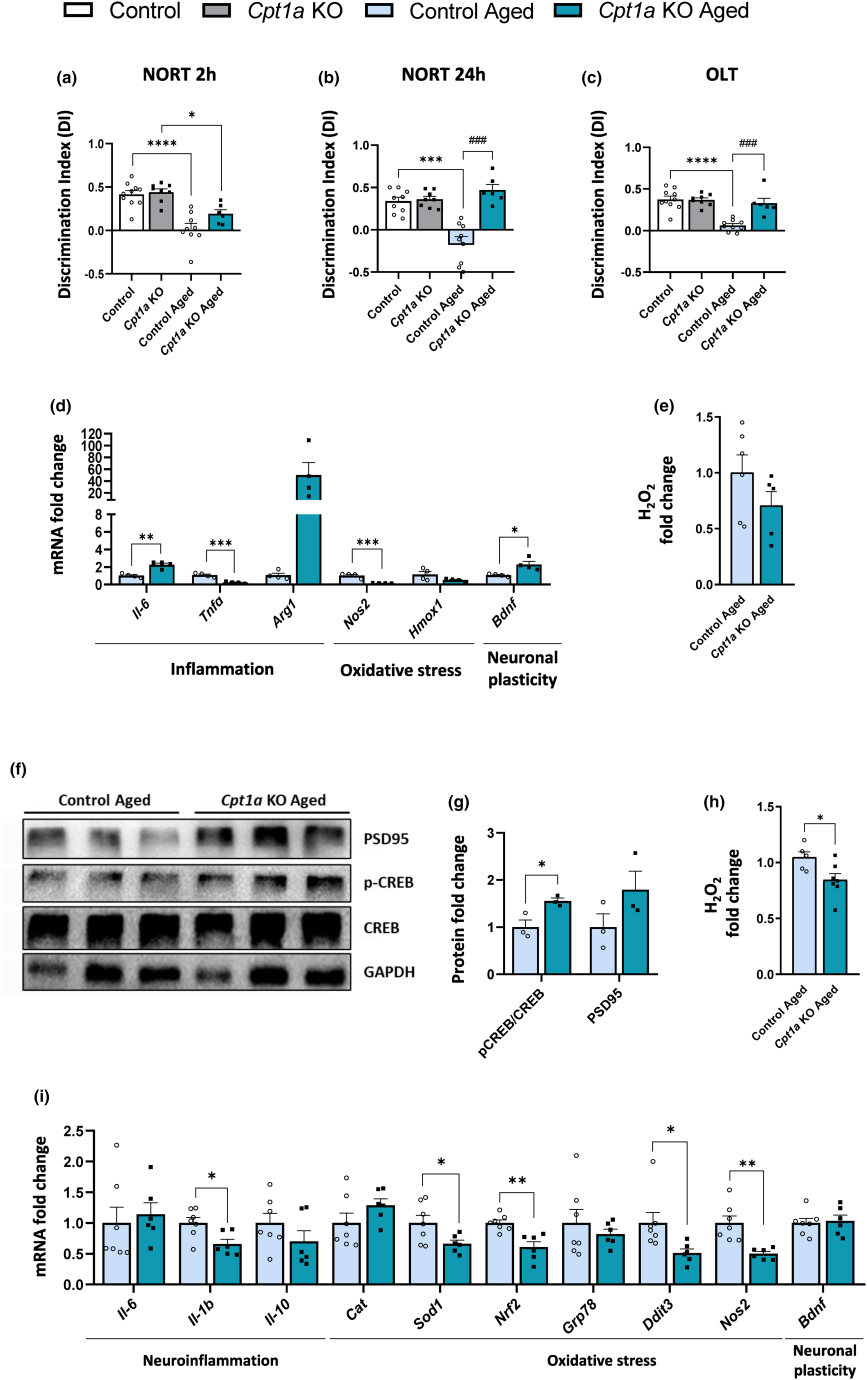
Cognition of aged *Cpt1a* KO and control male mice. (a,b) NORT at 2 and 24 h. (c) OLT results. (d) mRNA levels of genes related to inflammation, oxidative stress, and neuronal plasticity in the hippocampus of aged mice. (e) Analysis of ROS (H_2_O_2_ levels). (f,g) Proteins levels of genes involved in neuronal plasticity in the hippocampus of aged mice. (h) Analysis of ROS (H_2_O_2_ levels). (i) mRNA levels of genes related to neuroinflammation, oxidative stress, and neuronal plasticity in the hypothalamus of aged mice. ^# or^ *p < 0.05; ^## or^ **p < 0.01; ^### or^ ***p < 0.001; ****p < 0.0001 by two‐way ANOVA Sidak's (a–c), two‐tailed Student's *t*‐test (d–h). (*n* = 8–9 adult and *n* = 3–9 aged mice) *Indicates adult versus aged. ^#^Indicates control versus *Cpt1a* KO.

Recent studies revealed that AgRP neurons are not only critical regulators of food intake, but they are also involved in cognitive development (Salthouse, 2010). We analyzed the role of CPT1A in AgRP neurons on memory by using two different tests: NORT and OLT. Adult *Cpt1a* KO mice did not exhibit alterations in spatial and recognition memory (Figure 6a–c). However, aged *Cpt1a* KO mice displayed higher cognitive abilities compared with control mice. Despite no differences in DI at 2 h (Figure 6a), aged *Cpt1a* KO mice showed a 50% improvement in recognition memory 24 h after object replacement (Figure 6b). Similar results were obtained using the OLT, and an increase in spatial memory was observed in aged *Cpt1a* KO mice (Figure 6c). Interestingly, whereas control mice developed a normal decline in memory associated with age, aged *Cpt1a* KO mice maintained cognitive capacity without any signs of aging.

To further explore these cognition alterations, we analyzed the hippocampus, the main region of the brain involved in memory and learning. The hippocampal mRNA analysis of aged mice showed a reduction in the oxidative stress marker nitric oxide synthase 2 (*Nos2*), the inflammatory marker tumour necrosis factor alpha (*Tnfα*) and a trend (*p*‐value 0.09) to decrease the ROS content (H_2_O_2_ levels) in aged *Cpt1a* KO mice (Figure 6d,e). We also observed an increase in *Il‐6*, which plays a role in both inflammatory processes and neurogenesis in the brain (Erta et al., 2012). These mice also exhibited an increase in mRNA levels of bran‐derived neurotrophic factor (*Bdnf)*, a gene involved in neuroplasticity (Figure 6d). Consistent with these results, we also observed an increase in mRNA levels of postsynaptic density 95 (*Psd95*) gene and an increased ratio of phosphorylated cAMP response element‐binding (pCreb) to total Creb protein, suggesting an improvement in synaptic regulation and neuroplasticity (Figure 6f,g). It is also well known that hypothalamic inflammation is associated with the loss of cognitive function and behavior (Shoji et al., 2016). The hypothalamus of aged *Cpt1a* KO mice had reduced mRNA levels of several genes involved in inflammation (*Il‐1β*) and oxidative stress, such as superoxide dismutase type 1 (*Sod1*), nuclear factor erythroid 2 (*Nrf2*), DNA damage‐inducible transcript 3 (*Ddit3*), and *Nos2*. These results are supported by a reduction in the ROS content (H_2_O_2_ levels) (Figure 6h,i). Taken together, these findings confirm that CPT1A in AgRP neurons can modulate cognition during aging, and its deletion improves memory skills by increasing neuronal plasticity and reducing inflammation and oxidative stress in the hippocampus and hypothalamus.

## 4 DISCUSSION

In the present study, we investigated the specific function of CPT1A in hypothalamic AgRP neurons in response to physical and cognitive activities during aging. The main results of this study revealed that: (1) aged *Cpt1a* KO male mice exhibit a protection against aging decline accompanied by a reduction in IGF‐1 levels; (2) *Cpt1a* deletion in AgRP neurons improves physical performance and myofiber remodeling and this state is maintained during the progression of aging; and (3) aged mice lacking *Cpt1a* in AgRP neurons had improved cognition, increased neuroplasticity, and reduced oxidative stress and inflammatory markers in the hippocampus and hypothalamus.

### 4.1 *Cpt1a* KO mice exhibit protection against aging decline

We demonstrate a specific role for CPT1A in AgRP neurons of aged male mice. Aged *Cpt1a* KO male mice showed a significant reduction in the number of gray hairs compared with control mice. Hair graying is a natural feature associated with age. The gradual loss of pigmentation is related to a reduction in the enzymes involved in melanogenesis, antioxidant mechanisms, DNA repair, and antiapoptotic signals. It has been reported that IGF‐1 activates follicular proliferation, tissue remodeling, and the hair growth cycle, as well as follicular differentiation in transgenic mice (Li et al., 2014; Weger & Schlake, 2005). However, aged *Cpt1a* KO mice had decreased blood IGF‐1 levels, suggesting that factors other than IGF‐1 may contribute to the resistance to hair graying. On the contrary, a reduction in blood levels of IGF‐1, which is an important mediator of growth hormone actions, reportedly delays aging and significantly extends longevity (Junnila et al., 2013; K. Mao et al., 2018). This agrees with the extended longevity observed in aged *Cpt1a* KO mice. We previously observed that *Cpt1a* KO mice had reduced projections from AgRP neurons to the paraventricular nucleus (PVN) of the hypothalamus, enhancing the HPA axis and sympathetic tone. We also observed a decrease in blood levels of testosterone (Zagmutt et al., 2023). Since testosterone levels are mediated by the HPA, we cannot rule out that a drop in activation of PVN neurons from AgRP neurons may also modulate pituitary activity, reducing blood IGF‐1 levels in aged *Cpt1a* KO mice. Other factors, such as reduced food intake, observed in the *Cpt1a* KO male mice may also contribute to their extended lifespan (Redman et al., 2018; Zagmutt et al., 2023).

### 4.2 AgRP neurons require CPT1A to modulate exercise performance in adult and aged male mice

Exercise influences the synaptic activity of specific neuronal populations in the hypothalamus through the SNS and the secretion of myokines (Benite‐Ribeiro et al., 2016; He et al., 2018). Specifically, in the ARC nucleus, the activity of AgRP neurons change in response to exercise (Bunner et al., 2020; He et al., 2018). However, the effect of altered lipid metabolism in AgRP neurons on exercise performance was not yet defined. In the present study, we elucidated for the first time that CPT1A in AgRP neurons is necessary for the modulation of locomotion, motor coordination, and exploratory behavior in adult and aged mice. The improvement in physical activity could be due to the reduced body weight observed in the *Cpt1a* KO mice especially the performance in the treadmill, rotarod, and strength tests (Avila et al., 2017; Deacon, 2013b; J. H. Mao et al., 2015). However, the performance in the OFT and EPMT seems to be unaffected by body weight in rodents (Yoshizaki et al., 2020). In addition, the ability of aged mice lacking *Cpt1a* to develop the same level of exercise capacity as adult mice is remarkable because these animals do not show any features characteristic of decline due to aging (Graber et al., 2021). This may be a consequence of the reduced food intake observed in *Cpt1a* KO mice (Zagmutt et al., 2023), similar to that observed in mice under dietary restriction (Fahlström et al., 2012; Peters et al., 2022).

Furthermore, in the last decade, AgRP neurons have been associated with neuronal circuits of nonfeeding behavior, including those related to reward, anxiety, and compulsive behavior (Dietrich et al., 2015; Miletta et al., 2020). The results obtained from adult and aged *Cpt1a* KO male mice in the OFT and EPMT exhibited the same pattern of behavior as control mice, suggesting that the differences in physical activity were not related to anxiety. These findings reinforce the potential role of CPT1A, specifically in AgRP neurons, on the benefits of physical activity to prolong the aging process.

The *Cpt1a* KO mouse model displayed a relevant sexual dimorphism regarding feeding and energy expenditure (Zagmutt et al., 2023). Sexual differences were also observed in response to exercise activity. Interestingly, female mice lacking *Cpt1a* in AgRP neurons had no difference in locomotor activity and strength compared with their control littermates, highlighting the importance of sex and the requirement for more studies in females to understand the molecular changes involved in this regulation.

### 4.3 CPT1A in AgRP neurons regulates muscle mass and myofiber composition

Skeletal muscle is an adaptable tissue that can change its physiological, morphological, and metabolic properties in response to external stimuli. Adult and aged *Cpt1a* KO male mice exhibited a reduction in the GAS and QUA muscle mass. This reduction was not observed in other muscles, such as EDL and SOL muscles. In general, a decrease in muscle mass is associated with a reduction in muscle strength; however, no changes were showed in the strength capacity of adult *Cpt1a* KO mice. The decrease in muscle mass of adult and aged *Cpt1a* KO mice is tightly associated with a reduction in the CSA of the GAS and TA muscles and a significant increase in the number of type I muscle fibers that showed enhanced *Myh7* expression and suggesting a myofiber transition from type IIa to type I. Interestingly, our model seems to mimic the fiber remodeling described in aged humans, increasing type I CSA and reducing type II muscle fibers, although *Cpt1a* KO mice did not show the reduction of muscle strength observed in aged humans (Claflin et al., 2011; Trappe et al., 2003). Myofiber remodeling was not observed in muscles composed of specific types of muscle fiber, such as SOL (oxidative) and EDL (glycolytic) muscles, suggesting that only muscles with mixed fibers undergo remodeling.

The changes in muscle mass observed in adult and aged *Cpt1a* KO male mice could be explained by the involvement of certain hormonal and regulatory pathways, such as IGF‐1 and myostatin (Gdf8), which are involved in muscle protein synthesis (Giovannini et al., 2018). A crosstalk between IGF‐1 and Gdf8 signaling pathways has been reported, suggesting that Gdf8 is counteracting the signaling of IGF‐1 on muscle growth (Hennebry et al., 2017; Morissette et al., 2009). In addition, some pathways involved in protein degradation, including Fbxo32, and Murf‐1, may also, in part, overlap and be regulated by IGF‐1 signaling and Gdf8, reviewed in Yoshida & Delafontaine, 2020. Interactions between protein synthesis and degradation pathways provide a mechanism for IGF‐1 signaling to modulate the decreases in muscle mass in *Cpt1a* KO mice.

We also analyzed the development and plasticity of NMJs since they are essential for the function of skeletal muscle and a crucial factor in age‐related muscle weakness. Adult *Cpt1a* KO mice did not show any changes in the denervation marker *Ncam1* in GAS and TA muscles, whereas aged *Cpt1a* KO mice exhibit a reduction in *Ncam1* and *Musk* mRNA levels, which could suggest a lower muscle denervation. This could improve any reduction in the signs of muscle weakness during aging when the effect of muscle atrophy is more pronounced (Gonzalez‐Freire et al., 2014).

An increase in oxidative myofibers increases mitochondrial activity and function (Garcia et al., 2018; Shin et al., 2015). Aged mice lacking *Cpt1a* in AgRP neurons showed increased mitochondrial content in GAS. In agreement with other studies (Leduc‐Gaudet et al., 2021), no changes in the protein levels of mitochondrial dynamics' markers were observed. No differences were observed in the ROS production between control and *Cpt1a* KO mice and the lack of changes in pHSL levels could suggest an absence of lipolysis activation at the basal levels in GAS and TA. Altogether, these results suggest that the increase in the oxidative myofibers and mitochondrial content could support the ability of *Cpt1a* KO mice to improve physical activity during exercise.

The enhancement of physical activity in *Cpt1a* KO mice may be due to an increase in oxygen transport, which could improve the ability of these mice to continue exercising compared with the control mice. However, the GAS and TA muscles of *Cpt1a* KO mice had a reduction in mRNA levels of angiogenesis markers (*Vegf* and *Hif1a*), that could mimick the natural process that occurs during aging or may be a consequence of muscle mass loss. A reduction in mRNA levels of these genes influences oxygen transport and muscle cell function; however, this effect is not observed *Cpt1a* KO mice (Mehran Ghahramani, 2020). This fact highlights that other compensatory processes could be involved, such as the activation of SNS, changes in AgRP neuronal activity, or muscle mass reduction. Overall, although muscle strength is similar to control mice, the specific deletion of *Cpt1a* in AgRP neurons affects muscle mass and myofiber composition. These findings suggest that CPT1A in AgRP neurons could play a crucial role in myofiber remodeling.

### 4.4 *Cpt1a* ablation in AgRP neurons improves memory in aged male mice

Several studies have demonstrated that hypothalamic neurons are essential for diverse types of learning and memory (Domingos et al., 2013; Mavanji et al., 2017). Adult *Cpt1a* KO mice showed no differences in spatial and recognition memory with respect to control mice. However, aged *Cpt1a* KO mice exhibited better performance in the NORT and OLT, indicating improved cognition and memory compared with aged control mice. In addition, aged *Cpt1a* KO mice maintained cognitive abilities without any alterations during aging, when compared to aged control mice. During aging, neurons have increased oxidative stress, mitochondrial impairment, accumulation of damaged proteins, DNA lesions, and apoptosis, making them vulnerable to degeneration and loss of function (Liu et al., 2022; Mattson & Magnus, 2006). However, the hippocampal analysis of mRNA levels of aged *Cpt1a* KO mice indicates increased neuronal plasticity, as well as a reduced inflammation and a trend to reduce oxidative stress. In addition, the increased mRNA levels of arginase 1 (*Agr1)*, a marker of microglia M2, which have anti‐inflammatory and neuroprotective functions, support the beneficial effect of CPT1A deletion in aged mice (Colonna & Butovsky, 2017; Guo et al., 2022).

Similarly, aged *Cpt1a* KO mice exhibited reduced mRNA levels of the markers of neuroinflammation and the markers and content of oxidative stress in the hypothalamus compared with control mice. These data correlate with the improvement in cognitive abilities and suggest a potential role of *Cpt1a* in the regulation of memory, potentially both at the level of hippocampus and hypothalamus.

In summary, the present study uniquely demonstrates that a lack of CPT1A in AgRP neurons improves health in male mice. Notably, adult and aged *Cpt1a* KO male mice displayed improved physical activity without changes in anxiety‐related behavior. In addition, aged *Cpt1a* KO mice had significant improvements in memory and cognition. These results provide open new perspectives on the role of Cpt1a in AgRP neurons, and an opportunity to consider CPT1A as a potential antiaging candidate and therapeutic target for the treatment of diseases that would benefit from improvements in memory and physical activity.

## AUTHOR CONTRIBUTIONS

KI and DS contributed to study conception and design. KI, CGF, MMR, DS, MPB, RG, CSV, MP, MC, and PM were involved in data acquisition, data analysis, and interpretation. KI, DDB, LH, and DS contributed to manuscript writing and final review.

## CONFLICT OF INTEREST STATEMENT

The authors have no conflicts of interest to disclose.

## Supporting information


Appendix S1

## Data Availability

The data generated during the current study are available from the corresponding author on reasonable request.
