# Supplementary material for: Cpt1a silencing in AgRP neurons improves cognitive and physical capacity and promotes healthy aging in male mice
Source: Aging Cell. 2023 Nov 22;23(2):e14047. doi: 10.1111/acel.14047 (PMC10861206; doi:10.1111/acel.14047)
Supplement: Supplementary file 1 — Appendix S1 [file ACEL-23-e14047-s001.docx]

**Supplemental methods and experimental procedures**

**Weight test**

The weight test analyzes the muscle strength of the forelimbs. This test uses seven steel rings of different weight (8.2, 14.6, 24, 36.5, 51.2, 70.6, and 92.4 g) each containing a sponge ball linked to a collector to attach the rings. Mice were held by the middle base of the tail, allowing them to grasp the sponge with the ring. The final score was calculated following the criterion described by Deacon, 2013.

**Tissue collection from mice**

Blood samples were collected in heparinized tubes from a facial vein and the plasma was collected and stored at -20^o^C. Samples of different muscles were obtained and stored immediately at -80^o^C. For hematoxylin and eosin (H&E) staining, complete muscles were fixed in 10% formalin solution, neutral buffered (Sigma-Aldrich) for 24h and transferred to 1x phosphate buffered saline. To evaluate the myofiber composition, mice were anesthetized with a ketamine/xylazine solution and sacrificed by cervical dislocation. TA and GAS muscles were collected and embedded immediately in molds covered with Tissue-Tek Optimum Cutting Temperature reagent (Tissue-Tek). Embedded muscles were frozen by placing them into a pre-cooled solution of 2-methylbutane at -60^o^C for 5 min and stored at -80^o^C.

**Blood glucose and plasma hormone measurements**

Glucose levels were measured from blood samples obtained from the tail vein of 2 h-fasted mice. Measurements were made using a hand glucometer (Bayer Contour T). Several hormones including catecholamines (ImmuSmol), cortisone (Reagent Genie) and IGF-1 levels (R&D System) were measured in mouse plasma samples according to the manufacturer’s instructions.

**Immunostaining of muscle cross-sections**

Tissue sections were permeabilized in potassium phosphate buffered saline (KPBS) [0.9% NaCl, 52 mM K_2_HPO_4_ and 9.6 mM KH_2_PO_4_] containing 0.1% Triton X-100 for 10 min and blocked in a KPBS solution containing 0.1% Triton X-100, 2% BSA and 2% goat serum in a wet chamber for 1h. Slices were washed three times with KPBS for 5 min and incubated with primary antibody diluted in blocking buffer in a wet chamber at 4^o^C overnight. Muscle sections were washed three times with KPBS for 5 min, and then, incubated with secondary antibody diluted in blocking buffer for 2h at room temperature in a wet dark chamber. After the incubations, sections were covered with a drop of Fluoromount G (LabClinics) and placed under a cover slip. Fluorescent images were captured using a Leica DMI4000B microscope (Leica) and the number of muscle fibers and composition were analyzed using Fiji Image J software (Schindelin et al., 2012). A list of primary and secondary antibodies used is provided in Table S2.

**AgRP neuropeptide immunostaining**

AgRP neuropeptide immunostaining was performed on free-floating brain sections. Briefly, mice were intracardially perfused with PFA 4%. Frozen brains were embedded in OCT and 30-μm slices were obtained with a microtome (Leica SM2000R). Slices were permeabilized in potassium phosphate buffered saline (KPBS 0.1% Triton X-100), for 10 min and blocked for 1 h with blocking solution (KPBS containing 0.1% (v/v) Triton X-100, 3% (w/v) BSA and 2% (v/v) goat serum (Sigma-Aldrich, Cat# G9023). The slices were incubated with the AgRP antibody (Invitrogen, PA1-18414, dilution 1:1000) in blocking solution for 1 h at room temperature and ON at 4 °C. They were then washed 3 times for 10 min with KPBS containing 0.1% Triton X-100 before being incubated with goat anti-guinea pig IgG secondary antibody (red) at a dilution of 1:1000 in blocking solution for 2 h at room temperature and protected from the light. Finally, the samples were washed 3 times for 10 min with KPBS containing 0.1% Triton X-100 and mounted onto SuperFrost Plus slides (Thermo Fisher Scientific, Cat# J7800AMNT) with fluoromount-G containing hoechst 33342 and cover slipped. Sections containing the ARC nucleus were scanned with the Multiphoton Microscope Leica TCS SP8 MP. The LAXZ software was used to obtain a Z-stack of each sample at 40x magnification. The format was 1024 × 1024 pixels. AgRP signal in ARC sections was quantified using the Fiji ImageJ 1.33 (NIH; Bethesda, MD, USA).

**Protein extraction and western blotting**

Total protein was extracted from 50 mg of GAS, TA, hippocampus and hypothalamus sample and by adding 500 μl of lysis buffer (30 mM HEPES, 150 mM NaCl, 10% glycerol, 1% Triton X-100, 0.5% DOC, and a tablet containing 1x protease inhibitor and PhosSTOP phosphatase inhibitor). Tissue samples were homogenized, and the lysates were placed on a shaker at 4^o^C for 20 min. Samples were centrifuged at 13,000 rpm for 15 min and supernatants were carefully collected. Protein quantification was determined using the Pierce BCA protein assay (Thermo Scientific). Samples were prepared for electrophoresis by adding 6x loading buffer (375 mM Tris-HCl, pH 6.8; 9% SDS; 50% glycerol; 0.03% bromophenol blue and 9% β-mercaptoethanol) and distilled water to a final concentration of 1 μg/ml. Protein samples were denatured at 95^o^C for 5 min and 25 ml of protein samples were loaded onto a Criterion Empty Cassettes (BioRad). Protein electrophoresis was performed in the Criterion Gel Electrophoresis Cell System (BioRad). Electrophoresis was performed at a constant voltage of 120 V for 90 min. Proteins were then transferred to a 0.45 μm nitrocellulose membrane (BioRad) in a Criterion Blotter system with wire electrodes (BioRad) at 250 mA for 120 min. Membranes were washed three times with 1x washing buffer (10 mM Tris-HCl, pH 7.4, 150 mM NaCl, 0.1% Tween 20), blocked in 5% non-fat milk (ChemCruz) diluted in washing buffer and incubated with the appropriate primary antibody at 4^o^C overnight. The next day, membranes were washed three times with 1x washing buffer and incubated with peroxidase-conjugated secondary antibodies at room temperature for 2h in an orbital shaker. A list of primary and secondary antibodies and their dilutions are provided in Table S2. Immunodetection was performed by incubating membranes with enhanced chemiluminescence horseradish peroxidase substrate (ECL, Thermo Scientific) for 5 min. Band detection was performed using an Image Quant LAS 4000 Mini (GE Healthcare) and quantified by using Image J software (Version 1.8.0, Schneider, Rasband, and Eliceiri 2012).

**Total RNA extraction and quantitative real-time PCR (qRT-PCR)**

RNA was isolated from frozen samples using Trizol reagent (Sigma-Aldrich) according to the manufacturer's guidelines. RNA quality and quantity were measured using a Nanodrop 1000 spectrophotometer (Thermo Scientific). Purified RNA (500 ng) was reverse transcribed to complementary DNA (cDNA) using TaqMan reverse transcription reagents (Thermo Scientific), according to the manufacturer’s instructions with minor changes (both random hexamers and oligodT primers were used in the reaction). Two negative controls were included: (1) a pool of RNA samples without TaqMan polymerase enzyme and (2) all the reagents and water, without any RNA. The cDNA obtained was diluted to a concentration of 5 ng/μl. qRT-PCR was performed using the Power SYBR Green PCR Master Mix Reagent Kit (Roche) in a LightCycler 480 Instrument II (Roche) according to the manufacturer’s guidelines. mRNA levels from skeletal muscle were normalized to ribosomal protein L32 (*Rpl32*), and ribosomal protein L7 (*Rpl7*) was used as a standardization gene for the hypothalamus and glyceraldehyde-3-phosphate dehydrogenase (*Gapdh*) for the hippocampus. The primers used in this study are listed in Table S1.

**Measurement of reactive oxygen species (ROS)**

H_2_O_2_ levels were measured in different tissues using Amplex Red kit (Thermo Fisher) according to manufacturer’s instructions and as described in Irazoki et al., 2022.

**Bibliography**

Deacon, R. M. J. (2013). Measuring the strength of mice. *Journal of Visualized Experiments : JoVE*, *76*, 1–4. https://doi.org/10.3791/2610

Irazoki, A., Martinez-Vicente, M., Aparicio, P., Aris, C., Alibakhshi, E., Rubio-Valera, M., Castellanos, J., Lores, L., Palacín, M., Gumà, A., Zorzano, A., & Sebastián, D. (2022). Coordination of mitochondrial and lysosomal homeostasis mitigates inflammation and muscle atrophy during aging. *Aging Cell*, *21*(4), 1–16. https://doi.org/10.1111/acel.13583

Schindelin, J., Arganda-Carreras, I., Frise, E., Kaynig, V., Longair, M., Pietzsch, T., Preibisch, S., Rueden, C., Saalfeld, S., Schmid, B., Tinevez, J. Y., White, D. J., Hartenstein, V., Eliceiri, K., Tomancak, P., & Cardona, A. (2012). Fiji: An open-source platform for biological-image analysis. *Nature Methods*, *9*(7), 676–682. https://doi.org/10.1038/nmeth.2019

Schneider, C. A., Rasband, W. S., & Eliceiri, K. W. (2012). NIH Image to ImageJ: 25 years of image analysis. *Nature Methods*, *9*(7), 671–675. https://doi.org/10.1038/nmeth.2089

**
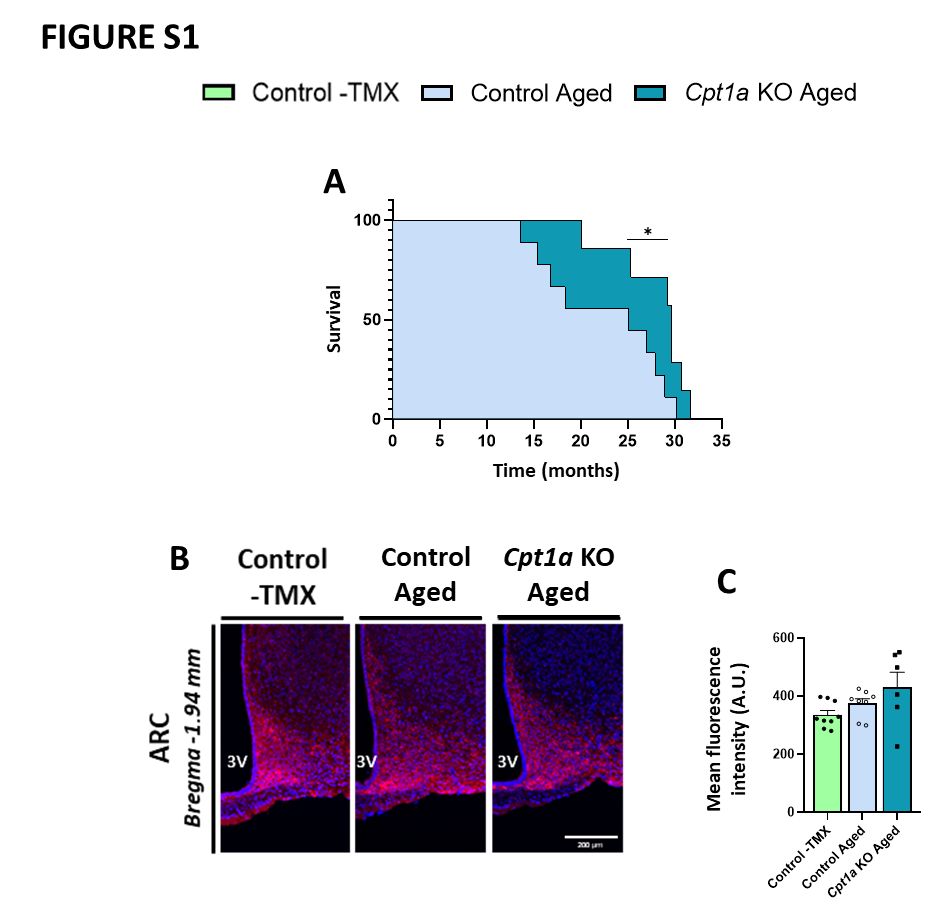
**

**Figure S1.** Lifespan of *Cpt1a* KO and control male mice. **(A)** Survival of mice (n=7-9).  **(B)** Representative images of AgRP neuropeptide immunodetection in the ARC of 22-month-old control mice non-induced with tamoxifen (Control-TMX), *Cpt1a* KO aged and control aged mice. Scale bar, 40 μm. **(C)** Quantification of AgRP neuropeptide fluorescent signal in the ARC of these mice groups. (n = 3). **P < 0.05.*

**
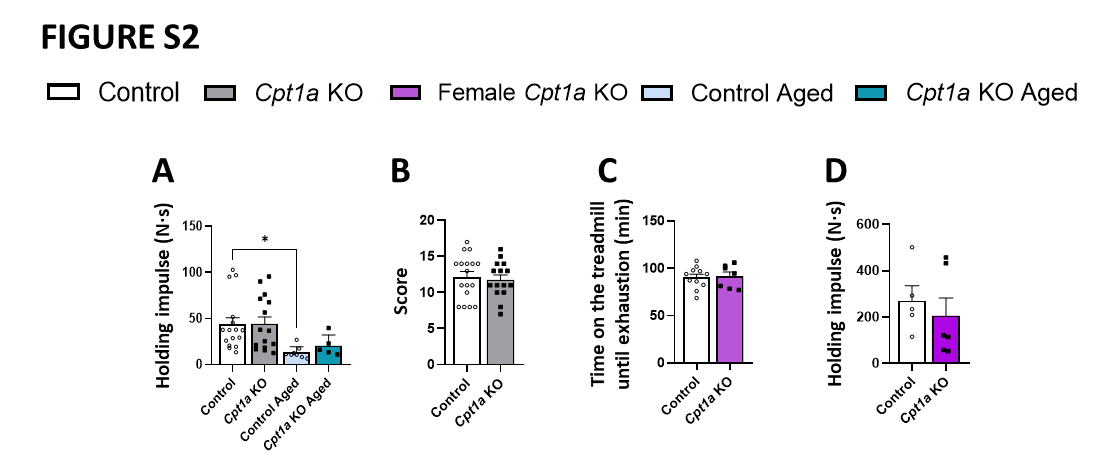
**

**Figure S2**. Behavioural performance of *Cpt1a* KO and control mice. **(A)** Kondziela’s inverted screen test results of adult and aged male mice. (**B)** Weight tests analysis of adult male mice. Treadmill exhaustion test **(C)** and Kondziela’s test **(D)** of adult female mice. **P < 0.05;* ^##^*P < 0.01; and* ^###^*P < 0.001* using two-way ANOVA Sidak’s multiple comparisons test (A) and two-tailed Student’s *t* test (B-D). (n=16-18 adult and n=6-9 aged male mice, n=6-8 adult female mice). *Indicates adult vs. aged. ^#^Indicates control vs. *Cpt1a* KO.

**
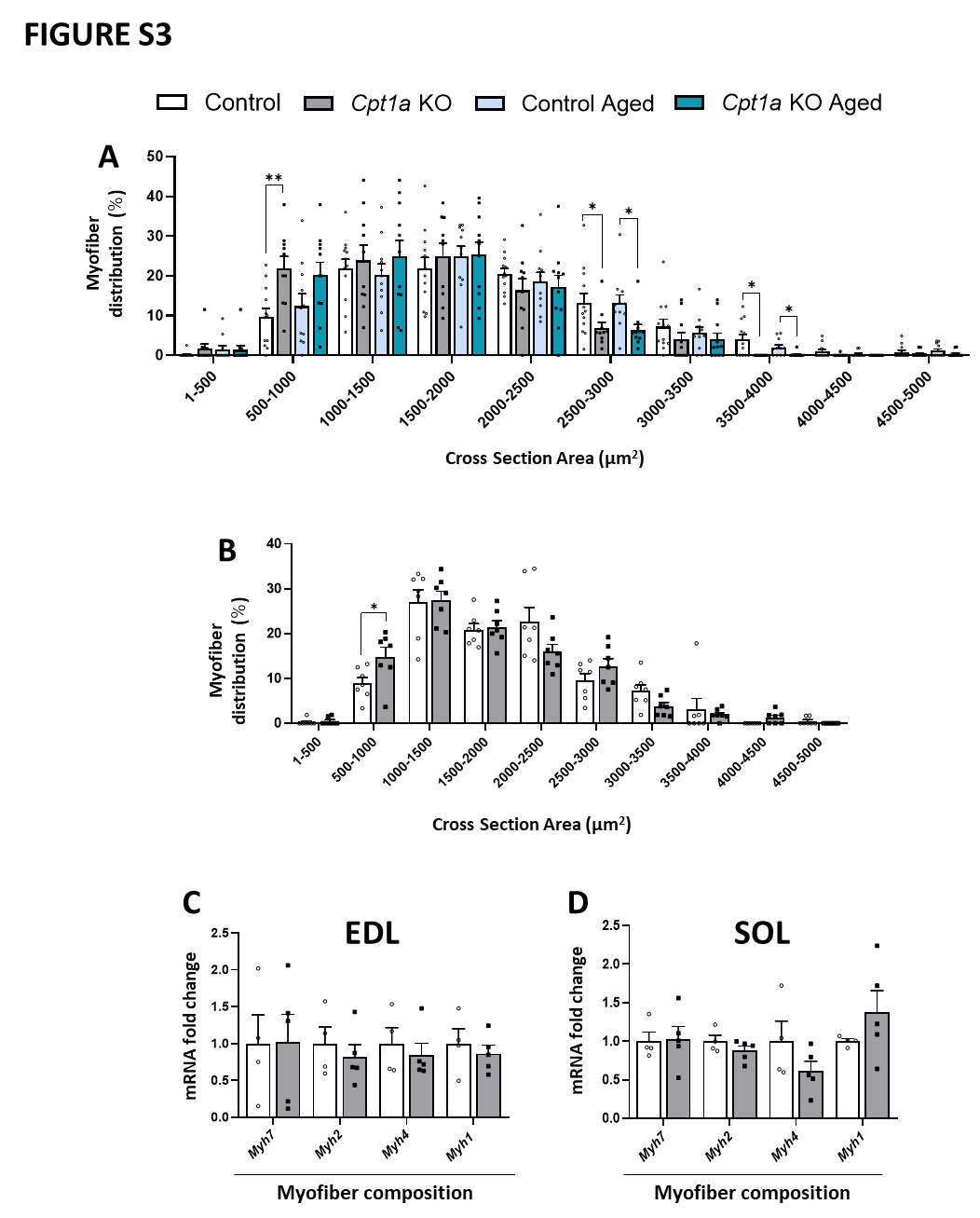
**

**Figure S3.** Analysis of myofiber composition of GAS, TA, EDL, and SOL muscles. Myofiber distribution of adult and aged mice in the GAS **(A)** and TA muscles **(B)**. Analysis of mRNA levels of genes related to myofiber composition in the EDL **(C)** and SOL muscles **(D)**. **P < 0.05; **P < 0.01* using two-way ANOVA Sidak’s multiple comparisons test (A) and two-tailed Student’s *t* test (B–D). (n=4-12 adult and n=6-9 aged male mice) *Indicates adult vs. aged mice.

**
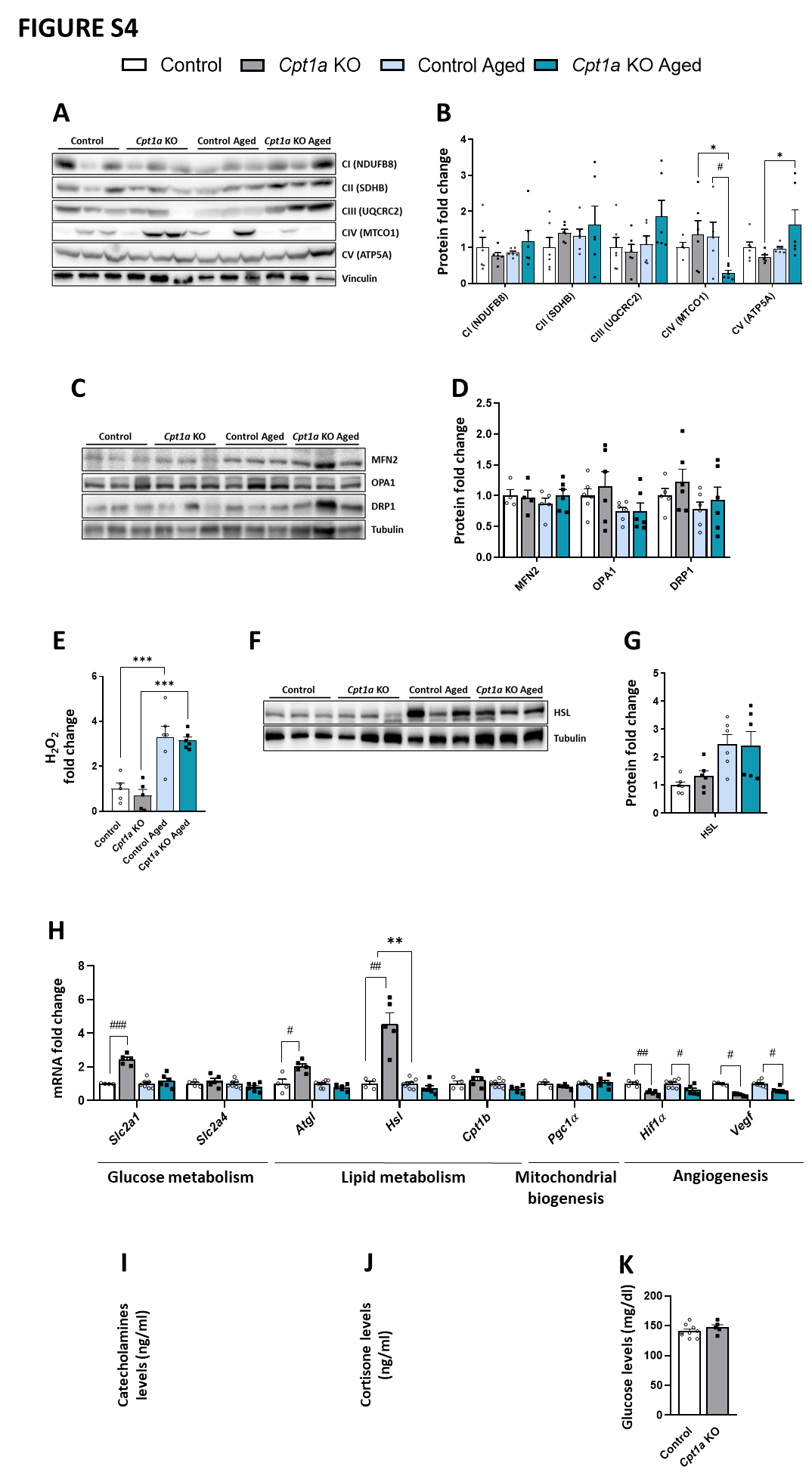
**

**Figure S4**. Analysis of mitochondrial content, mitochondrial dynamics, metabolism and angiogenesis in TA muscle. Analysis of the mitochondrial content **(A-B).** Representative western blot of the mitochondrial OXPHOS proteins **(A).** Quantification **(B).** Analysis of mitochondrial dynamics **(C-D).** Representative western blot of the mitochondrial dynamics protein markers **(C).** Quantification **(D). (E)** Analysis of ROS (H_2_O_2_ levels). **(F-G)** Analysis of the protein marker of lypolisis. Representative western blot of total HSL protein **(F).** Quantification respect to the tubulin **(G). (H)** mRNA levels of genes related to glucose, lipid metabolism and angiogenesis in the TA muscle. Plasma catecholamine levels **(I)**, plasma cortisone levels **(J)** and plasma glucose levels **(K)** in adult and aged mice. ^# or^**P < 0.05;* ^## or^***P < 0.01;* ^### or^***** *P < 0.001;* using two-tailed Student’s *t* test (I and K) and two-way ANOVA Sidak’s multiple comparisons (A–H and J). (n= 4-9 adult and =6-9 aged mice) *Indicates adult vs. aged. ^#^Indicates control vs. *Cpt1a* KO.

**Tables**

**Supplemental Table 1**. List of primers sequences used in this project.

| ****Primer ID**** | ****Forward (5’→3’)**** | ****Reverse (5’→3’)**** |
| --- | --- | --- |
| ****Rpl7**** | **TCGCAGAGTTGAAGGTGAAG** | **GCCTGTACTCCTTGTGATAGTG** |
| ****Rpl32**** | **GCTGCCATCTGTTTTACGG** | **TGACTGGTGCCTGATGAACT** |
| ****AgRP CreERT2**** | CAGATACCATCATCTCTCCC | CCTTAAACTCGCCCATATATGTGG |
| ****AgRP CreERT2 control**** | - | GCTCTACTTCATCGCATTCCTTG |
| ****Arg1**** | **CTCCAAGCCAAAGTCCTTAGAG** | **AGGAGCTGTCATTAGGGACATC** |
| ****Atgl**** | **TGACCATCTGCCTTCCAGA** | **TGTAGGTGGCGCAAGACA** |
| ****Bdnf**** | **AGTCTCCAGGACAGCAAAGC** | **TGCAACCGAAGTATGAAATAACC** |
| ****Cat**** | **GTGCATGCATGACAACCAG** | **TGAAGCGTTTCACATCTACAGC** |
| ****Cpt1a Flox**** | CAGGATCCCTTTGAGCAGCAG | CAAAGTGGCCCCTAAGGCTAC |
| ****Cpt1b**** | **TGCCTTTACATCGTCTCCAA** | **GGCTCCAGGGTTCAGAAAGT** |
| ****mt-Cytc**** | **CTACAAGACGCCACAT** | **GAGAGGGGAGAGCAAT** |
| ****Ddit3**** | **CCCTGCCTTTCACCTTGG** | **CCGCTCGTTCTCCTGCTC** |
| ****Drp1**** | **CTGGATCACGGGACAAGG** | GTTGCCTGTTGTTGGTTCCT |
| ****Fbxo32**** | **AGTGAGGACCGGCTACTGTG** | **GATCAAACGCTTGCGAATCT** |
| ****Gapdh**** | **ACTCCACTCACGGCAAATTC** | **TCTCCATGGTGGTGAAGACA** |
| ****Gdf8**** | **TGGCCATGATGATCTTGCTGTAA** | **CCTTGACTTCTAAAAAGGGATTCA** |
| ****Grp78**** | **ACTTGGGGACCACCTATTCCT** | ATCGCCAATCAGACGCTCC |
| ****Hif1α**** | **AACAGAATGGAACGGAGCAA** | **TTCACAATCGTAACTGGTCAGC** |
| ****Hmox1**** | **CATAGGCGGCCAGGAACATT** | **TCCTCGTTCCAGAATGCAGC** |
| ****Hsl**** | **GCGCTGGAGGAGTGTTTTT** | **CGCTCTCCAGTTGAACCAAG** |
| ****Il-1β**** | **GCCCATCCTCTGTGACTCAT** | **AGGCCACAGGTATTTTGTCG** |
| ****Il-6**** | **GATGGATGCTACCAAACTG** | **CCAGGTAGCTATGGTACTCCAGAA** |
| ****Il-10**** | **GGTTGCCAAGCCTTATCGGA** | **ACCTGCTCCACTGCCTTGCT** |
| ****Mfn2**** | **CATTCTTGTGTCGGAGGAG** | **AAGGAGAGGGCGATGAGTCT** |
| ****Murf1**** | **TGACATCTACAAGCAGGAGTGC** | **TCGTCTTCGTGTTCCTTGC** |
| ****Musk**** | **TACAGAGGGGAGGTGTGTGA** | **TCCCGGTAGGAGGTGTTGAA** |
| ****Myh-1**** | **GAGGGACAGTTCATCGATAGCAA** | **TGCTAATGCCCTAATGCTAATG** |
| ****Myh-2**** | **AGGCGGCTGAGGAGCACGTA** | **GCGGCACAAGCAGCGTTGG** |
| ****Myh-4**** | **CACCTGGACGATGCTCTCAGA** | **GCTCTTGCTCGGCCACTCT** |
| ****Myh-7**** | **CGCATCAAGGAGCTCACC** | **CTGCAGCCGCAGTAGGTT** |
| ****Ncam1**** | **CCCAGCCAAGGAGAAATCAG** | **TGGCGTTGTAGATGGTGAGG** |
| ****Nos2**** | **CAGCTGGGCTGTACAAACCTT** | **GCATTGGAAGTGAAGCGTTTC** |
| ****Nrf2**** | **CAGAAGGAACAGGAGAAGGC** | **TTTGGGAATGTGGGCAACCT** |
| ****Opa1**** | **TTCTGAGGCCCTTCTCTTGT** | **TGACTGTTGCTCGAAATGC** |
| ****Pgc1α**** | **GAAAGGGCCAAACAGAGAGA** | **GTAAATCACACGGCGCTCTT** |
| ****Slc2a1**** | **AGCTTGATCACCTCGTAGGC** | **TTACAGCGCGTCCGTTCT** |
| ****Slc2a4**** | GATGACCGTGGCTCTGCT | **GCTCTGCCACAATGAACCA** |
| ****Sdha**** | TACTACAGCCCCAAGTCT | TGGACCCATCTTCTATGC |
| ****Sod1**** | **CAGGACCTCATTTTAATCCTCAC** | **CCCAGGTCTCCAACATGC** |
| ****Tnfα**** | **CTGTAGCCCACGTCGTAGC** | **TTTGAGATCCATGCCGTTG** |
| ****Vegf**** | **AAGACAGAACAAAGCCAGAAAA** | **AGAGGTCTGGTTCCCGAAA** |

**Supplemental Table 2**. List of primary and secondary antibodies used in western blot and immunohistochemistry.

| ****Antibodies**** | ****Dilution**** | ****Source**** |
| --- | --- | --- |
| **Anti-rabbit Dystrophin** | **1:1000** | **Abcam, #ab152777** |
| **Anti-mouse Myh2** | **1:300** | **DSHB, #2F7** |
| **Anti-mouse Myh4** | **1:300** | **DSHB, #10F5** |
| **Anti-mouse Myh7** | **1:300** | **DSHB, #BA-F8** |
| **Anti-mouse Gapdh** | **1:2000** | **Abcam, #ab8245** |
| **Anti-rabbit pCreb (Ser^133^)** | **1:1000** | **Cell Signaling, #9198** |
| **Anti-rabbit Creb** | **1:1000** | **Cell Signaling, #4820** |
| **Anti-rabbit Psd95** | **1:1000** | **Abcam, #ab18258** |
| **Anti-mouse Mitofusin2** | **1:1000** | **Abcam #ab56889** |
| **Anti-mouse OPA1** | **1:1000** | **Abcam #ab157457** |
| **Anti-mouse Drp1** | **1:1000** | **BD transduction Lab #611112** |
| **Anti-mouse OXPHOS** | **1:2000** | **MitoSciences #MS6040** |
| **Anti-rabbit HSL** | **1:1000** | **Cell Signaling #4107** |
| **Anti-rabbit Phospho-HSL (Ser565)** | **1:1000** | **Cell Signaling #4137** |
| **Anti-mouse alpha Tubulin** | **1:1000** | **Abcam #ab7291** |
| **Anti-mouse Vinculin** | **1:2000** | **Abcam #ab18058** |
| **Anti-mouse AgRP** | **1:1000** | **Invitrogen # PA1-18414** |
| **Anti-guinea pig IgG1, Alexa Fluor 568** | **1:1000** | **ABACAM # ab175714** |
| **Goat anti-rabbit IgG, Alexa Fluor 405** | **1:1000** | **Invitrogen, #A31556** |
| **Goat anti-mouse IgM, Alexa Fluor 488** | **1:1000** | **Invitrogen, #A21042** |
| **Goat anti-mouse IgG1, Alexa Fluor 568** | **1:1000** | **Invitrogen, #A21124** |
| **Sheep anti-mouse, HRP-conjugated** | **1:1000** | **Abcam, #ab8245** |
| **Goat anti-rabbit IgG, HRP-conjugated** | **1:1000** | **Cell Signaling, #9272** |
| **Donkey anti-mouse HRP-conjugated** | **1:10000** | **Jackson ImmunoResearch #715-035-150** |
| **Donkey anti-rabbit HRP-conjugated** | **1:10000** | **Jackson ImmunoResearch #711-035-152** |
